# Truly-optimized PWR lattice for innovative soluble-boron-free small modular reactor

**DOI:** 10.1038/s41598-021-92350-5

**Published:** 2021-06-18

**Authors:** Xuan Ha Nguyen, Seongdong Jang, Yonghee Kim

**Affiliations:** Department of Nuclear and Quantum Engineering, Korea Advanced Institute of Science and Technology (KAIST), 291 Daehak-ro, Yuseong-gu, Daejeon, 34141 Republic of Korea

**Keywords:** Nuclear energy, Nuclear physics

## Abstract

A novel re-optimization of fuel assembly and new innovative burnable absorber (BA) concepts are investigated in this paper to pursue a high-performance soluble-boron-free (SBF) small modular reactor (SMR), named autonomous transportable on-demand reactor module (ATOM). A truly optimized PWR (TOP) lattice concept has been introduced to maximize the neutron economy while enhancing the inherent safety of an SBF pressurized water reactor. For an SBF SMR design, the 3-D centrally-shielded BA (CSBA) design is utilized and another innovative 3-D BA called disk-type BA (DiBA) is proposed in this study. Both CSBA and DiBA designs are investigated in terms of material, spatial self-shielding effects, and thermo-mechanical properties. A low-leakage two-batch fuel management is optimized for both conventional and TOP-based SBF ATOM cores. A combination of CSBA and DiBA is introduced to achieve a very small reactivity swing (< 1000 pcm) as well as a long cycle length and high fuel burnup. For the SBF ATOM core, safety parameters are evaluated and the moderator temperature coefficient is shown to remain sufficiently and similarly negative throughout the whole cycle. It is demonstrated that the small excess reactivity can be well managed by mechanical shim rods with a marginal increase in the local power peaking, and a cold-zero shutdown is possible with a pseudo checker-board control rod pattern. In addition, a thermal–hydraulic-coupled neutronic analysis of the ATOM core is discussed.

## Introduction

The soluble-boron-free (SBF) pressurized water reactors (PWRs) have not been practical since it was proposed a long time ago^1^. It is largely due to the lack of successful burnable absorber (BA) designs that can achieve sufficiently small excess reactivity without compromising neutronic and safety performances. Recently, the SBF operation has been renewed, particularly on water-cooled small modular reactors (SMRs) with various BA designs^2–7^. However, their competitiveness should be further improved to compete with Gen-III LWRs and other types of SMR^8,9^, even though SBF SMRs have many advantages such as system simplicity, less corrosion, less liquid wastes, short construction time, passively-enhanced safety, etc.^10^. In addition, it is difficult to achieve the cold shutdown in the SBF operation as temperature defect is significant with highly negative temperature coefficients, while the number of control rod (CR) is limited. To assure cold shutdown in SMRs, the conventional checker-board pattern can hardly be used, instead the fraction of rodded FAs should be substantially increased^2^, leading to a complicated CR driving mechanism due to space shortage at the top of the reactor vessel. Overall, for practical SBF SMRs, the core performances must be enhanced and a simplified CR pattern is strongly required.

Most of the current water-cooled SMRs utilize the commercial PWR fuel assembly (FA), which is neutronically designed under soluble boron condition^2,3,5,7^. The hydrogen-to-uranium (HTU) ratio is in a significantly under-moderated region for a slightly negative moderator temperature coefficient (MTC). This assures a level of the inherent safety of the reactor. However, it noticeably compromises the neutron economy. In an SBF system, the possibility of positive MTC is no longer a concern due to absence of soluble boron. Hence, the FA for an SBF system has more room for neutronic re-optimization and its neutron economy can be enhanced by increasing the HTU^11^. In addition, a higher HTU results in a less negative temperature coefficient. Consequently, the temperature defect between hot-full-power (HFP) and cold-zero-power (CZP) conditions becomes smaller, requiring smaller CR worth.

This paper is concerned with a PWR-type SMR, named Autonomous Transportable on-demand Reactor Module (ATOM), which is currently under development at Korea Advanced Institute of Science and Technology (KAIST) in Korea^7,12^. The standard FA design was first adopted in the early ATOM cores. For the latest ATOM core, a newly optimized FA design, so-called TOP (Truly-Optimized PWR) lattice, has been developed to improve both neutron economy and safety of the core. A preliminary investigation on the TOP-based FA design was presented in Ref. 13. For a successful SBF operation, it is crucially important to minimize the excess reactivity with BAs without compromising the reactor performances so that the criticality can be well controlled by using weak control rods. For the current ATOM, a new combination of 3-D innovative BAs is proposed based on centrally-shielded BA (CSBA) and disk-type BA (DiBA)^7,13^ to assure sufficiently small excess reactivity. Details of the TOP design, BA design and loading, CR arrangements, and two-batch ATOM core optimizations are presented in this paper.

In this work, the Monte Carlo Serpent 2^14^ with the nuclear library ENDF/B-VII.1 is mainly used for the neutronics calculations. All associated uncertainties of the Serpent results are given with one-sigma confidence interval. Meanwhile, a multi-physics core calculation is also performed by a two-step Serpent-COREDAX^15^ system.

## Innovations for soluble-boron-free small modular reactor

### The truly-optimized PWR lattice

**Figure 1:**
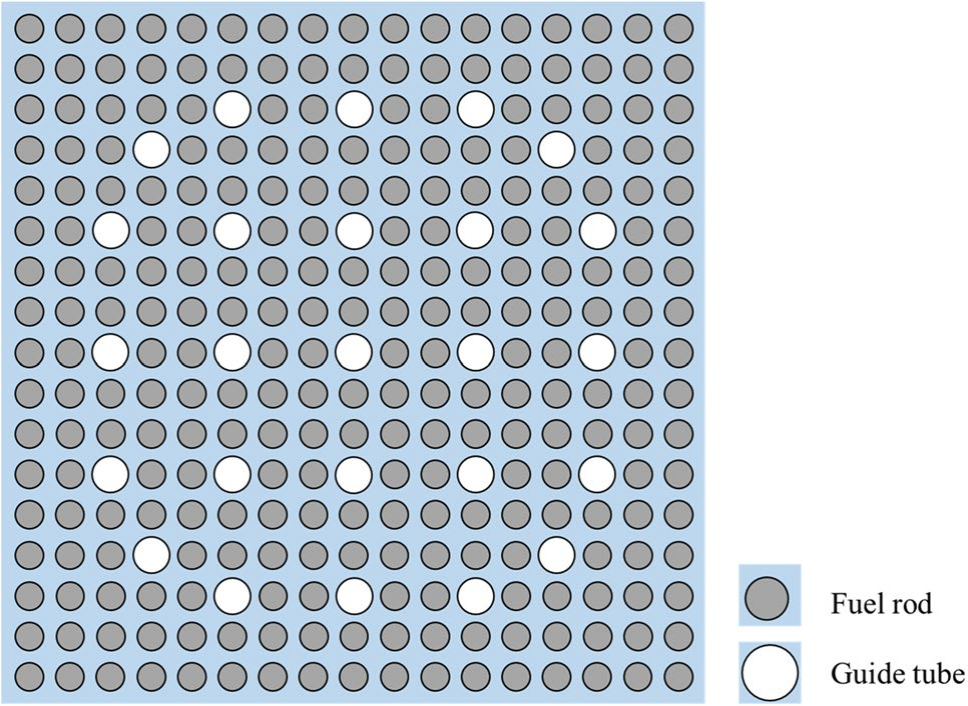
17 × 17 commercial PWR lattice.

Figure 1 depicts the typical 17 × 17 PWR FA and its detailed dimensions and specifications are given in Table 1^16^. The average coolant and fuel temperatures are taken from typical PWR designs. It should be noted that the HTU ratio is about 4.1. It is mentioned that other commercial FA designs such as Korean APR1400^17^ have a similar HTU ratio.

**Table 1:** Reference 17 × 17 FA design specification.

Parameter	Value
Fuel lattice	17 × 17
No. of fuel rods / guide tubes	264/25
Fuel material / pellet radius	UO_2_/0.40958 cm
Uranium enrichment	5 w/o
Cladding inner / outer radii	0.41873/ 0.47600 cm
Reference pin pitch	1.2623 cm
Reference FA pitch	21.6038 cm
Reference HTU ratio	4.1
Coolant / fuel temperatures	575 K / 900 K
Coolant density	0.72266 g/cm^3^

Based on the typical FA design, there are two ways to enhance the HTU ratio to improve the neutron economy. The first one is to reduce the fuel rod diameter or the number of fuel rod per FA while preserving the FA and active core size. However, fuel inventory per FA can be reduced significantly in this case, consequently the cycle length may be reduced unacceptable, which outweighs the improved neutron economy due to enhanced moderation^11^. In addition, a new fuel pellet needs to be developed in the case of the reduced fuel diameter. On the other hand, the second way is to enlarge the pin pitch while preserving the fuel rod dimension. Thus, the FA and active core sizes become slightly bigger in the radial direction. In this paper, the modification on the pin pitch is concerned to assess the TOP design, as thermal and mechanical performances of the standard fuel rod are rather well validated under the standard PWR conditions. Moreover, for the existing PWRs, the first approach is possible as the core size is fixed. Meanwhile, for the new reactor design like ATOM, both of the two options are possible. However, the second way is adopted to minimize the fuel rod design changes.

In order to select a TOP design for an SBF SMR, several objectives must be taken into account:

- Maximization of the neutron economy.
- Sufficiently & appropriately negative MTC and fuel temperature coefficient (FTC).
- Minimization of the temperature defect for improved CR worth margin.
- Maintenance of the clad-to-coolant heat transfer characteristics.
- Acceptable size of FA.

**Figure 2:**
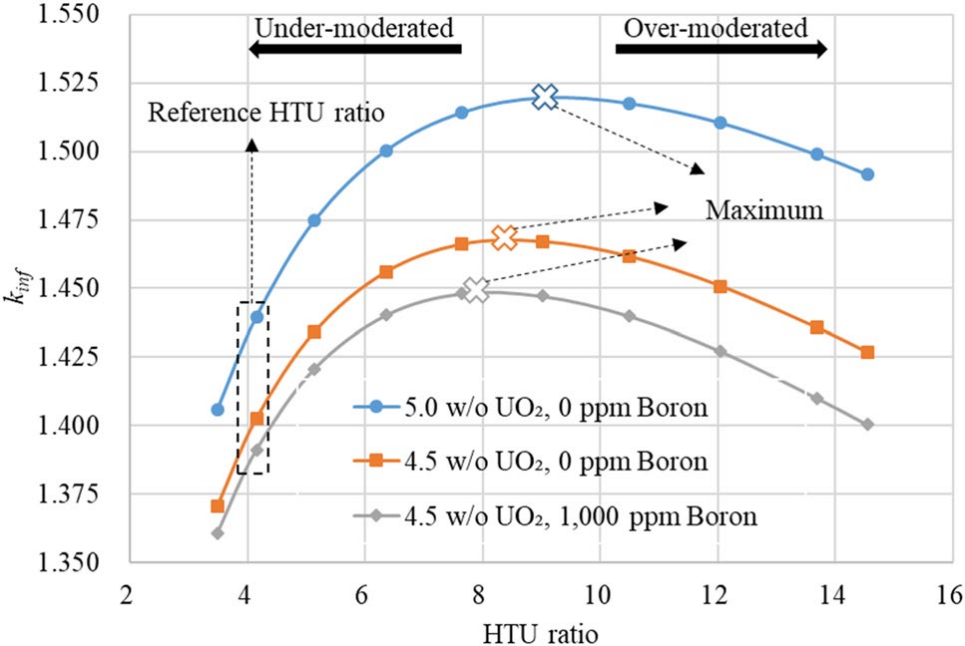
The infinite multiplication factor with respect to HTU value.

To find an optimal pitch for the typical FA design, a parametric study has been performed for a 2-dimensional infinite array of the FA using the Serpent 2 code^14^. The pin pitch is adjusted to obtain various HTU ratios and Fig. 2 shows the infinite multiplication factor (*k*_*inf*_) with respect to the HTU ratio at zero burnup for two fuel enrichments and boron concentrations at the hot full power (HFP) condition. In the Serpent calculations, 300 active and 100 inactive cycles are used with 100,000 histories per cycle, resulting in about 5.0 pcm uncertainty of the *k*_*inf*_ values.

One can clearly notice in Fig. 2 that the typical FA design is quite under-moderated due to the soluble-boron and the neutron economy is far from the optimal condition for the two commercial enrichment regardless of the boron. For the 5.0 w/o case, the optimal HTU ratio is about 9.0. Consequently, the *k*_*inf*_ value can be increased quite substantially by increasing the HTU value in an SBF core. In the commercial PWRs, the fuel lattice is quite under-moderated mainly due to potentially positive MTC at hot-zero-power (HZP)-BOC condition requiring a high boron concentration^17^. In the TOP approach, the HTU value should be determined such that the MTC should be sufficiently and appropriately negative over the whole cycle with an acceptable FA size, and the fuel utilization should be enhanced. It is important to recall that the HTU ratio should not be too close to the optimal condition in Fig. 2 since the MTC will be quite small or close to zero.

**Figure 3:**
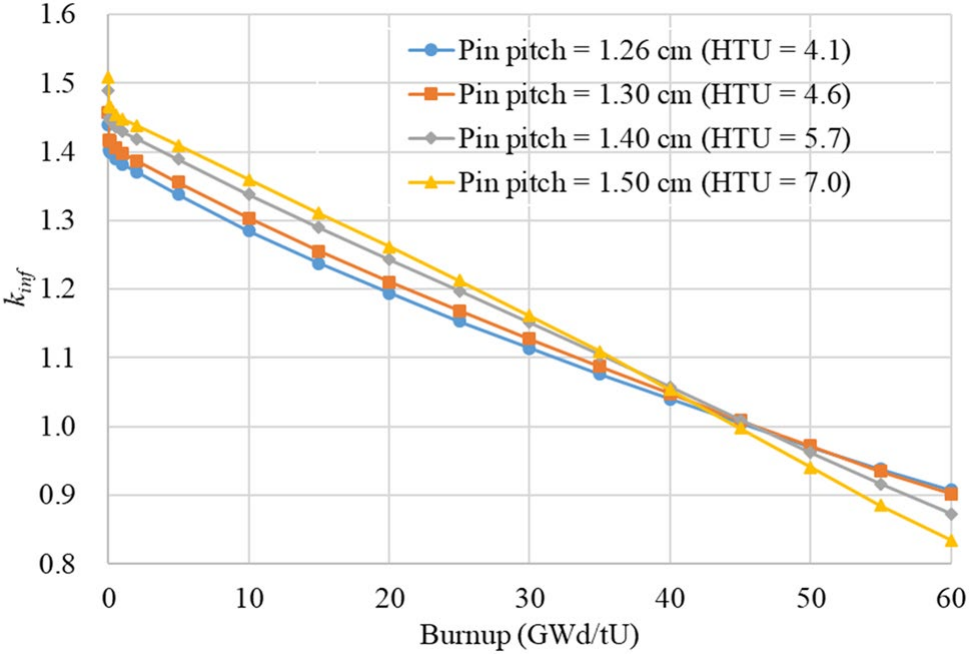
The burnup-dependent k_inf_ behavior with respect to various HTU values.

To quantify benefit of the TOP design in terms of fuel burnup, non-poisonous FA with 4.95 w/o U was depleted using Serpent and the burnup-dependent *k*_*inf*_ values are plotted in Fig. 3 for a few HTU values. One standard deviation of the *k*_*inf*_ values in Fig. 3 is about 10 pcm. In the FA depletion, specific power density of the ATOM core is used, i.e., 26 W/gU^7^. One can notice that the BOC *k*_*inf*_ clearly increases when HTU increases from 4.1 to 7.0. However, after a certain burnup, the behavior of *k*_*inf*_ is inversed, which is due to smaller buildup of Pu-239 and higher fission product poisoning in a softer spectrum.

In order to investigate the cycle length and discharge burnup for a two-batch fuel management (FM), a linear reactivity model^18^ is used and the results are given in Table 2, in which it is assumed that neutron leakage is 7,000 pcm. One can see that the cycle length and discharge burnup increase noticeably with a slight increase in the HTU ratio from the reference one. It should be noted that the fuel burnup is rather maximized with the HTU ratio of ~ 5.7, about 3% higher than the reference case, and then it even decreases with further increasing HTU ratio beyond 5.7. One also observed that the FA size increases by about 10% for HTU = 5.7, which will lead to a ~ 10% bigger core size. Therefore, the HTU can be increased up to ~ 5.7 for the TOP design if the 10% larger FA is acceptable in the core design.

**Table 2:** Cycle length and discharge burnup for various HTU ratios.

HTU ratio	Pin pitch (cm)	Discharge burnup (GWd/tU)	Cycle length (days)	FA pitch (cm)
4.1	1.26	46.4	913	21.60382
4.6	1.30	47.5	936	22.24472
5.7	1.40	47.7	940	23.94472
7.0	1.50	46.3	911	25.64472

**Figure 4:**
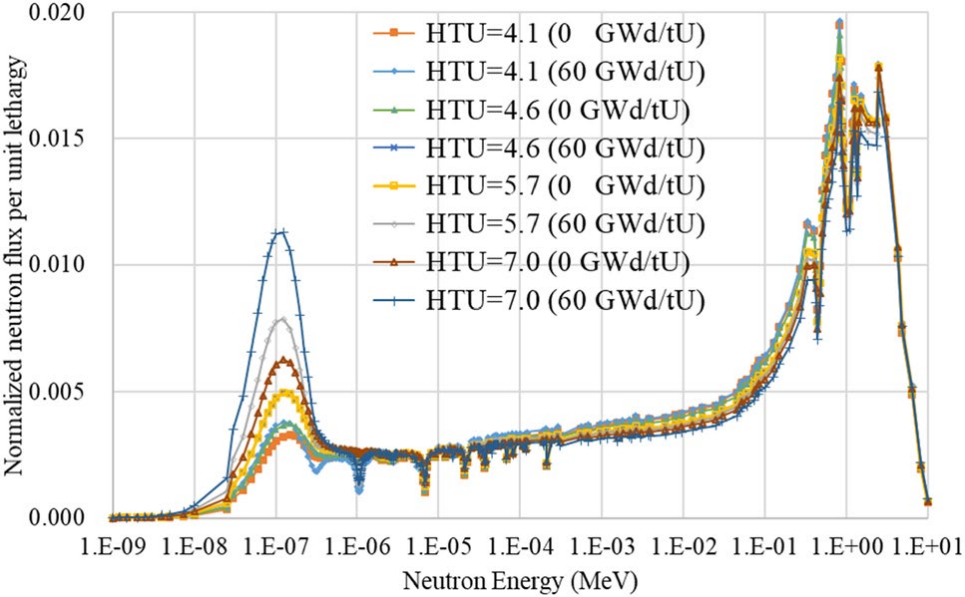
Neutron spectrum with respect to HTU ratio and burnup.

Figure 4 shows a neutron spectrum comparison for several HTU ratios at two burnup points. It can be seen that the spectrum becomes softened with bigger HTU ratio. In particular, the spectrum is much softer with HTU = 7.0 and this enhances fission product poisoning leading to a lower fuel burnup. In addition, it is important to note that 100% mixed-oxide (MOX) core is likely to be feasible as the significantly softer spectrum of the TOP design, an enhanced-moderation design, can resolve problems caused by the spectrum hardening due to Pu isotopes^19–21^.

**Figure 5:**
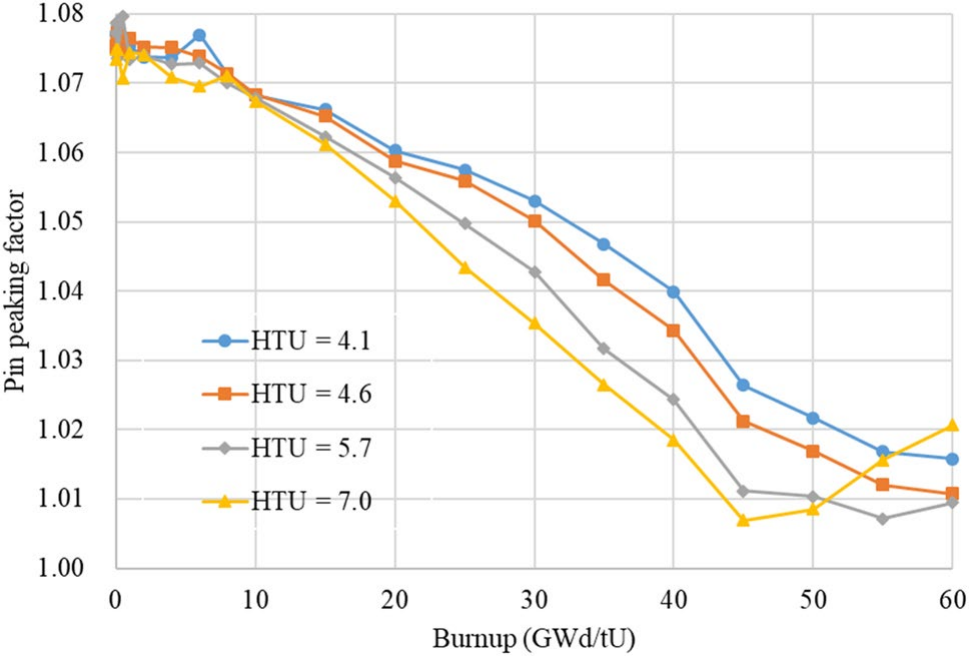
Burnup-dependent PPK with respect to HTU value.

In Fig. 5, power peaking factor (PPF) in the FAs for various HTU values is plotted as a function of burnup. One can notice that PPF decreases with burnup for all cases and the maximum value is about 1.08 at 0 GWd/tU. It is also observed that a larger HTU ratio results in a similar or slightly smaller PPK than that of the reference one regardless of burnup. It is because the impact of water-filled guide tube on PPF is less significant as the neutron spectrum becomes softer with a higher HTU ratio. The associated uncertainty of the PPF is about 0.5% in this analysis.

The most important parameters for the inherent safety of the core are temperature coefficients, FTC and MTC, which must be always negative at any condition. Nevertheless, too much negative temperature coefficients are not always preferable, such as a strongly negative MTC at EOC condition (e. g., − 63 pcm/K) results in a large control worth requirement^7^. In addition, a less negative FTC reduces the deviation of inlet coolant during an autonomous operation^22^. It is recommended that the MTC should be around -30.0 pcm/K and the FTC should be about − 2.0 pcm/K for a successful passive frequency operation^22^. The FTC and MTC values for various HTU ratios at HFP condition are listed in Table 3. One can notice that temperature coefficients become less negative with increased HTU ratio due to the softener neutron spectrum, while they are more negative with burnup due to Pu-239 and fission poisoning buildups. The optimal HTU ratio for autonomous operation is around 5.7 as the FTC is about −2 pcm/K and the MTC is around −30 pcm/K. The associated uncertainties of FTC and MTC are 0.14 pcm/K and 0.8 pcm/K, respectively. It is assumed that temperature coefficients are linear functions of temperature in this evaluation.

**Table 3:** Temperature coefficients (pcm/K) for various HTUs at HFP.

HTU	MTC @ 0 GWd/tU		MTC @ 60 GWd/tU	
	Wo/ BA	W/ BA	Wo/ BA	W/ BA
4.1	− 23.5	− 33.4	− 58.33	− 61.7
4.6	− 22.2	− 31.0	− 50.16	− 54.9
5.7	− 15.4	− 23.9	− 33.47	− 36.9
7.0	− 8.60	− 16.9	− 16.8	− 18.6

HTU	FTC @ 0 GWd/tU		FTC @ 60 GWd/tU	
	Wo/ BA	W/ BA	Wo/ BA	W/ BA
4.1	− 1.91	− 2.97	− 3.75	− 3.87
4.6	− 1.74	− 2.79	− 3.50	− 3.64
5.7	− 1.52	− 2.38	− 2.95	− 3.12
7.0	− 1.30	− 2.04	− 2.52	− 2.66

The reactivity difference between HFP and CZP conditions is defined as temperature defect, which is compensated by CR insertion to obtain CZP condition. Temperature defects from HZP to HFP for various HTU ratios and fuel burnups are tabulated in Table 4. As expected, it decreases significantly with increased HTU ratio since temperature coefficients are smaller with a higher HTU ratio as shown in Table 3. It is advantageous that a smaller shutdown rod worth is required for a larger HTU ratio. In addition, CR radius can be enlarged with a higher HTU ratio to enhance the CR worth further. The associated uncertainty of the temperature defect is about 12 pcm.

**Table 4:** Temperature defect with respect to HTU ratio and burnup.

Case	@ 0 GWd/tU	@ 60 GWd/tU
HTU = 4.1	4941 pcm	7943 pcm
HTU = 4.6	4490 pcm	7045 pcm
HTU = 5.7	3359 pcm	4484 pcm
HTU = 7.0	2302 pcm	1833 pcm

Overall, an optimal HTU ratio to meet aforementioned TOP goals is about 5.7. The use of TOP lattice maximizes the cycle length, reduces the temperature defect for an enhanced cold shutdown margin, and provides sufficient temperature coefficients for an autonomous operation, while assuring the inherent safety of the core. The pin pitch corresponding to 5.7 HTU ratio is 1.40 cm, which is then adopted in the two-batch ATOM core. One should note that the equivalent diameter of the TOP-based ATOM core is 224 cm, which is about 10% higher than that with the standard ATOM design^7^.

In the selected HTU ratio for the TOP design, the coolant flow area is increased by ~ 30% and it should affect thermal-hydraulics designs of the fuel assembly and its impacts are dependent on design choice among several options. Since this work is largely concerned with the neutronic attributes of the TOP concept, it is assumed that the average coolant speed remains unchanged and the coolant inlet temperature is appropriately increased for a given coolant temperature rise. In this case, the FA thermal-hydraulics will be quite similar to the conventional one, while the balance of the plant design should be accordingly modified. Meanwhile, the coolant speed can be reduced significantly in the TOP design if the same coolant temperature rise is adopted, and the core pressure drop should decrease a lot. However, in this approach, the thermal-hydraulics will be quite different due to a slower coolant flow, e.g., the critical heat flux can be lowered if other design measures are not introduced. Therefore, the thermal–hydraulic design for the TOP lattice needs to be optimized for the given plant system.

### Innovative burnable absorbers for the TOP ATOM core

To obtain a very small excess reactivity in the SBF ATOM core without compromising the core performances, a new innovative 3-D BA design, centrally-shielded burnable absorber (CSBA), is utilized^7^. In the CSBA design, gadolinia (Gd_2_O_3_) is loaded into the central region of the fuel pellet in the spherical form, which provides the strongest self-shielding effect of the BA, resulting in slow gadolinium depletion.

A recent 3-D multi-physics study^23^ demonstrated that the effective stress at the interface between CSBA balls and fuel is very acceptable, while the maximum temperature of the CSBA-loaded fuel pellet is comparable to that of the conventional one. In addition, the effect of asymmetric power distribution due to neighboring effect on the fuel temperature is relatively small, about 15 K in terms of peak temperature and subsequent thermal expansion and stress are hardly changed. Moreover, the material and experimental studies for the CSBA-loaded fuel are currently under-investigation and several preliminary outcomes are available at references^24,25^.

**Figure 6:**
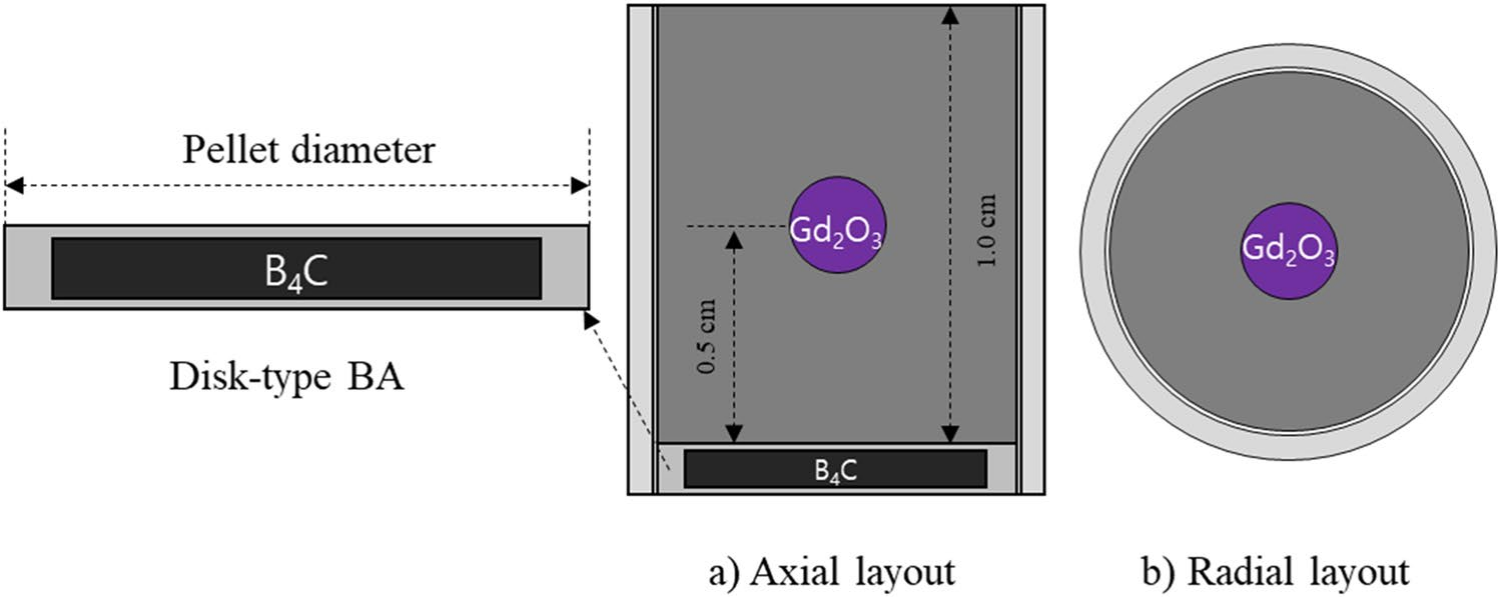
DiBA and CSBA-loaded fuel pellet.

Gadolinia is an effective and well-proven BA material in the nuclear technology. However, it is disadvantageous in that the residual gadolinium isotopes, e. g., Gd-158 and Gd-160, result in noticeable reactivity penalty. In addition, a simple 1-ball CSBA design is more favorable than 2-ball and 3-ball designs in terms of fabrication and quality control. Therefore, for a more flexible reactivity control, B_4_C is additionally used as the second BA material to reduce the residual gadolinium for enhanced neutron economy. In this work, B_4_C is used in the form of disk-type burnable absorber (DiBA), which was recently proposed^13^. A combination of the two BA designs is shown in Fig. 6. The B_4_C disk is cladded with Zr-4 with axial cladding thickness of 0.04 mm, while the outer diameter of radial cladding is the same as pellet diameter. The number of DiBA is identical to the number of fuel pellets per fuel rod, so-called 1P1D (1 pellet 1 DiBA) option.

**Figure 7:**
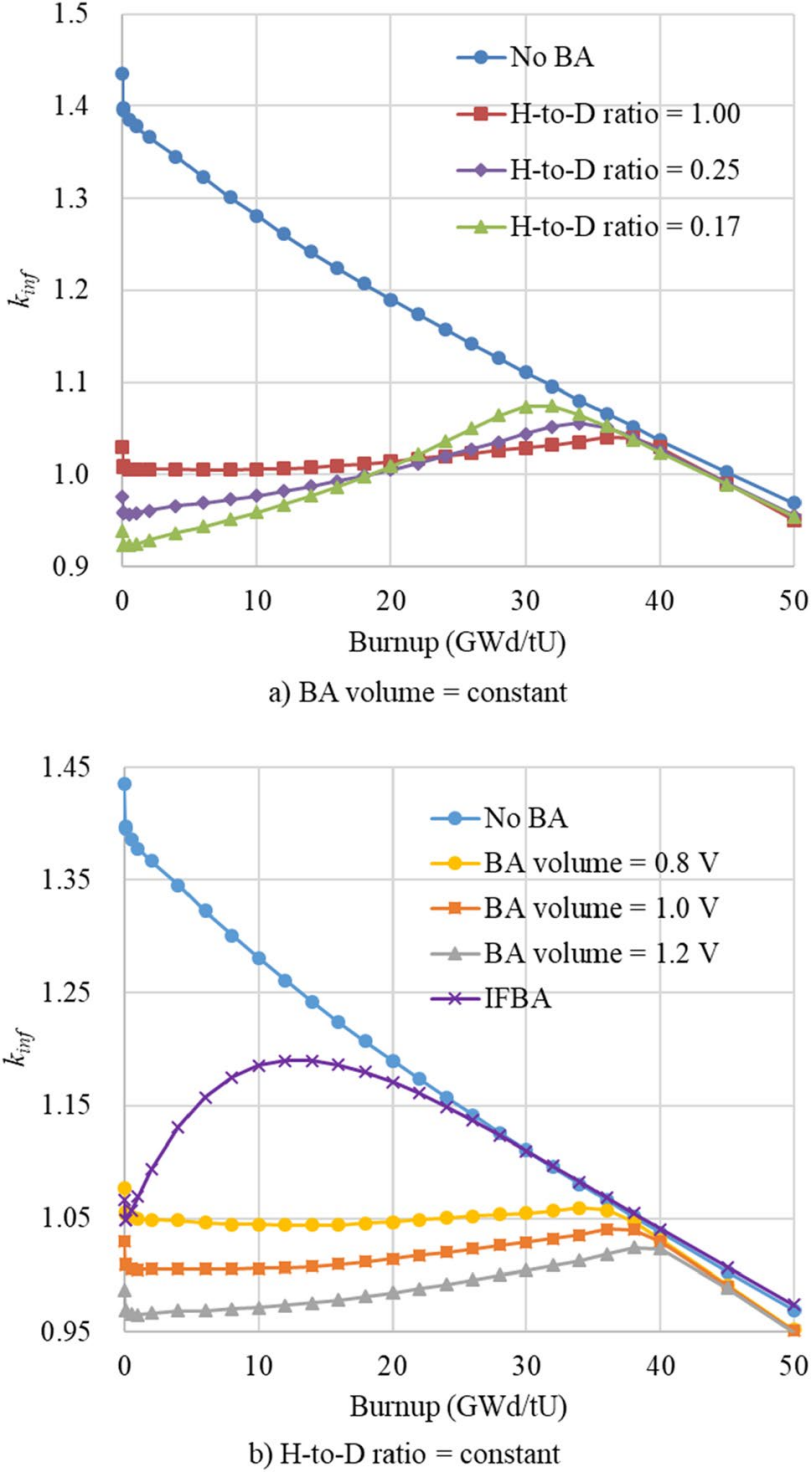
Infinite multiplication factor of the DiBA-loaded FA with respect to BA volume and H-to-D ratio.

Advantage of DiBA is that the self-shielding effect can be flexibly adjusted by controlling both the height-to-diameter (H-to-D) ratio and the volume of the BA disk as shown in Fig. 7, in which neutronic calculations of 17 × 17 lattice are performed with the Serpent 2 code. The number of active and inactive cycles are 200 and 100, respectively, with 100,000 histories. The associated uncertainty of the infinite multiplication factor is about 7.0 pcm. One should note that the amount of B_4_C should be limited so that the internal rod pressure due to fission gas and helium gas from B-10 depletion should not exceed the upper limit. In this study, the B_4_C volume is adjusted so that B-10 loading should be 0.09 mg B-10 per mm pellet, which is typical of the conventional IFBA design^26^. A 90% enriched B-10 is utilized to minimize the DiBA volume.

Figure 7b compares the *k*_*inf*_ value for B_4_C DiBA designs and ZrB_2_ 112-IFBA design^16^. The *k*_*inf*_ values of the DiBA case with 0.016 cm^3^ B_4_C (BA volume = 0.8 V) and the IFBA case at the fresh condition are adjusted to be the same for a consistent neutronic comparison. It can be seen clearly that the *k*_*inf*_ of IFBA design increases significantly after Xenon equilibrium. It is because boron in the IFBA design quickly burns out as the ZrB_2_ coating layer exposes largely to the neutron flux. The *k*_*inf*_ of the IFBA design decreases linearly and follows the no-BA case once boron depletes completely at 20 GWd/tU. On the other hand, boron in the DiBA case depletes rather slow as the *k*_*inf*_ stays around 1.05 until 30 GWd/tU. It is due to the H-to-D ratio close to 1.0, which minimizes the exposure of the BA to neutron flux. However, the reactivity penalty of the DiBA design is higher than that of the IFBA as a large amount of BA is necessary to hold down the excess reactivity throughout the cycle.

**Table 5:** Major design specifications of the TOP-based ATOM core.

Parameter	Value
Thermal power	450 MWth
FA type, number of FA	17 × 17, 69
Fuel material, enrichment	UO_2_, 4.95 w/o
Pellet radius	0.40958 cm
Pin pitch (cm)	1.40 cm
Radial reflectors	SS-304
BA designs	1-ball CSBA, 1P1D DiBA
Gd_2_O_3_ density in CSBA	99% TD*
B-10 enrichment in DiBA	90 w/o
No. of feed/burned FAs	35/34

*TD: theoretical density.

## The SBF two-batch ATOM core

### The ATOM core design

**Figure 8:**
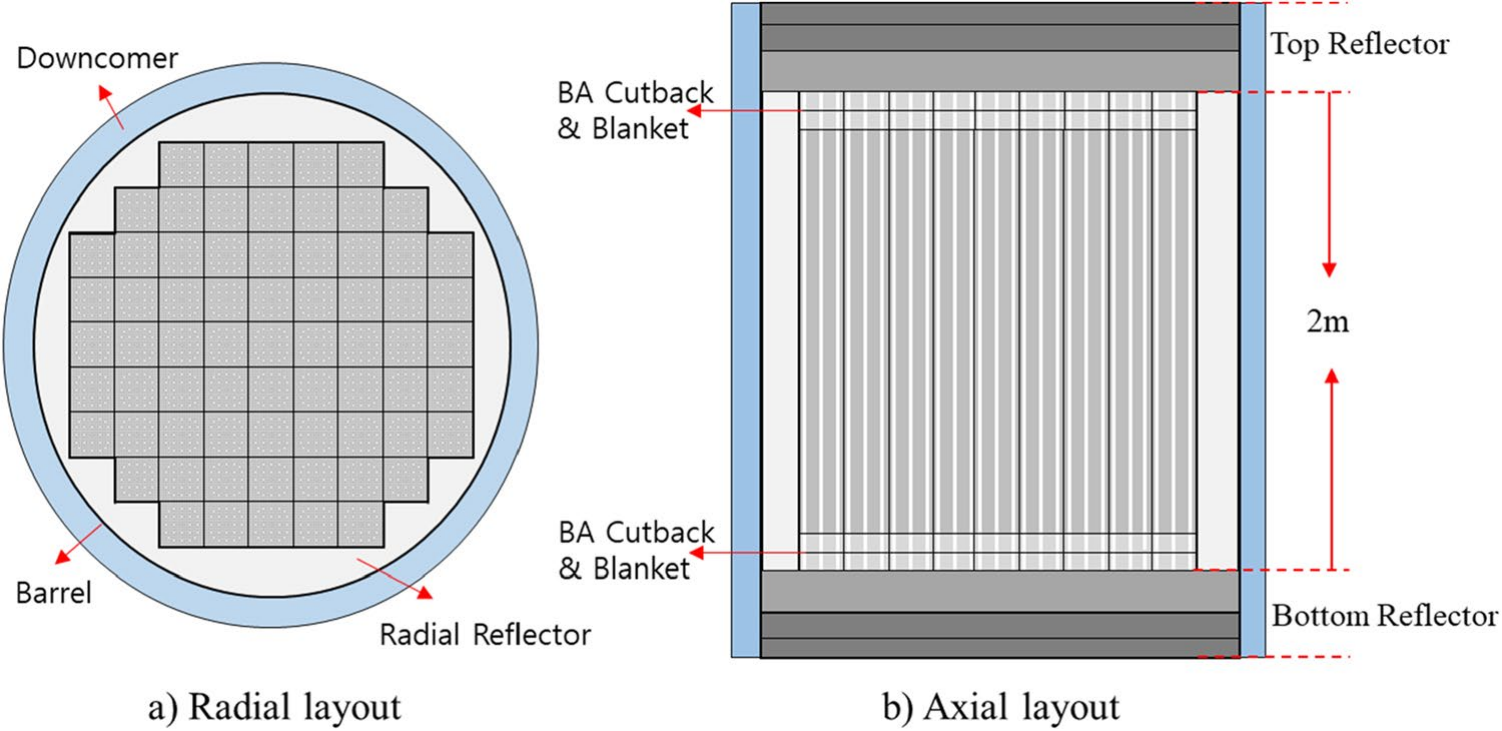
The radial and axial ATOM core layouts.

Major design parameters and cross-sectional views of the ATOM core are presented in Table 5 and Fig. 8, respectively. Thermal power of the core is set to 450 MWth and the active core consists of 69 TOP-based FAs with 2 m active height. The active core is surrounded by a stainless steel reflector and the axial reflector composition is adopted from the typical PWR design. The enrichment of UO_2_ fuel is 4.95 w/o with 95.5% theoretical density. At the top and bottom of the active core, 5 cm blanket with 2.0 w/o enrichment and 5 cm BA cutback with enrichment of 4.95 w/o are placed, as shown in Fig. 8. To achieve a reactivity swing smaller than 1,000 pcm, both 1-ball CSBA design and 1P1D DiBA designs are adopted^7^, as shown in Fig. 6. The reactivity swing is defined as the maximum excess reactivity during the cycle after xenon equilibrium in this work. Note that each FA has a single CSBA and DiBA design for simplicity. An accident-tolerant-fuel (ATF) cladding, Cr15Al-coated Zir-4, is also utilized for improved safety of the reactor^18^, in which the Cr15Al coating thickness is 30 micron.

**Figure 9:**
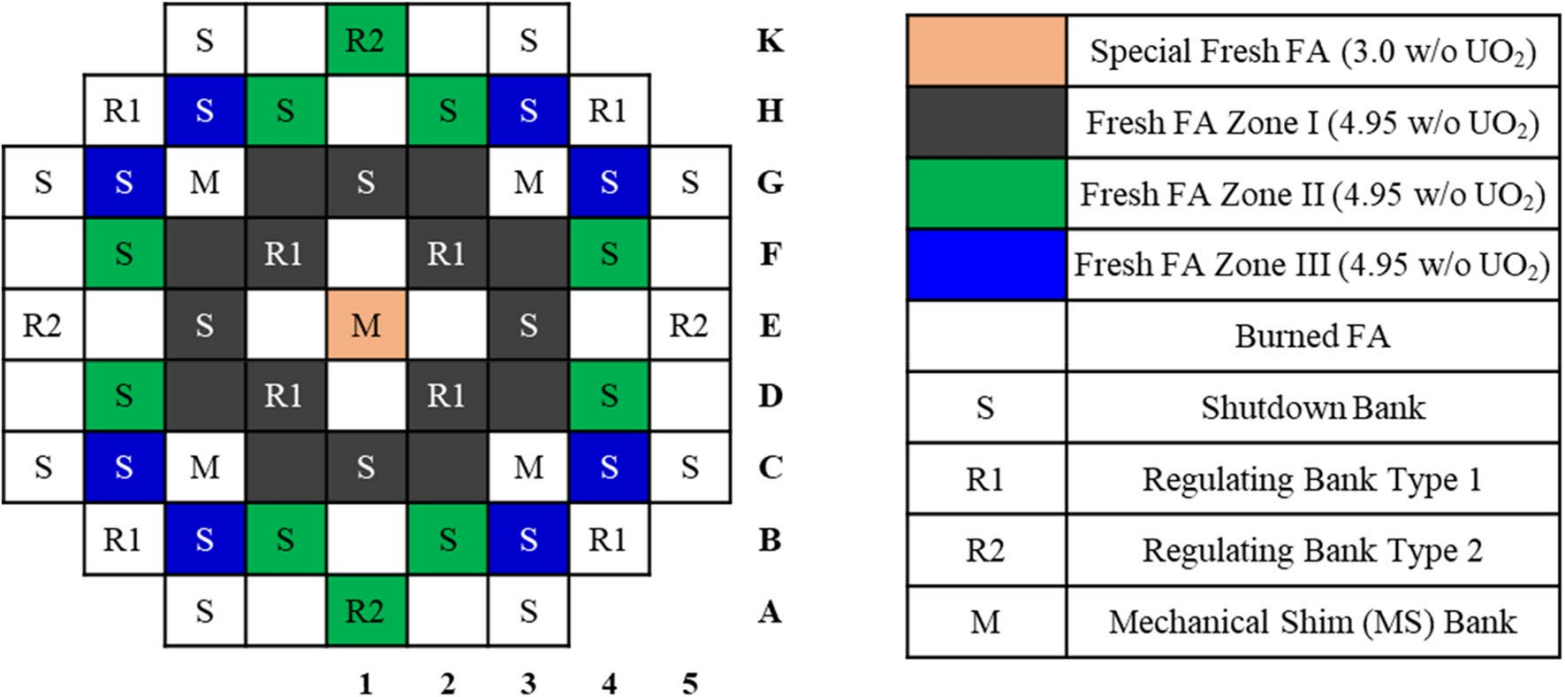
Radial zone-wise fuel loading and CR pattern.

A two-batch fuel management (FM) is adopted to maximize both cycle length and fuel burnup in this work and the fuel loading pattern is shown in Fig. 9. For enhanced neutron economy, the core utilizes an in-then-out fuel shuffling scheme as shown in Fig. 9 and Table 6. Most of fresh FAs are loaded in the inner regions while burned FAs are located mostly in the peripheral regions to minimize the neutron leakage. Several burned FAs are placed in the inner core for a flat radial power profile. The central FA is separately treated with an enrichment of 3.0 w/o and the number of standard feed FAs is 34 with 4.95 w/o UO_2_. Due to the number of the fresh FAs, the core is rotationally symmetric. Except for the center FA, the fresh FAs are radially divided into three zones: Zone I, zone II, and zone III. There are 16 fresh FAs in the inner region, zone I, where the power is the highest in general. These FAs are then shuffled into outermost positions introducing the lowest leakage as they are highly burned after their first cycle. On the other hand, 4 fresh FAs in the outermost zone, zone III, are shuffled into the center positions surrounding the center FA to reduce the power peaking here. Meanwhile, the other four FAs in zone III are reloaded in the outer positions, F1 and E5. Most of fresh FAs in zone II follow in-then-out shuffling scheme, in which they are shuffled to the neighboring outer positions, for example from F4 to F5, whereas fresh FAs in K1 position are reloaded in to E4.

Figure 9 also shows the CR pattern of the core and the CR design parameters are presented in Table 7. It is noteworthy that a pseudo checker-board pattern is adopted in the current ATOM core. The CR pattern comprises 28 shutdown control element assemblies (CEAs), 12 regulating CEAs, and 5 mechanical shim (MS) CEAs, respectively. In the TOP FA design, the CR is designed to be a little bigger than that in the standard one^16^ for improved CR worth. A B_4_C with 90 w/o B-10 is used as the material for the shutdown rod, while the regulating rod type 1 is based on natural B_4_C absorber. The MS is supposed to be quite weak and an Inconel 625-clad Mn (manganese) was chosen as the absorber of MS. The use of Inconel 625-clad Mn is to minimize power distortion during criticality control with the MS and to achieve a long lifetime of the MS absorber. In addition, the regulating rod type 2 is identical to the MS design to minimize the power peaking in the middle of large power maneuvering such as reactor startup. In order to achieve the MS worth similar to the burnup reactivity swing, the Mn rod radius and Inconel-625 thickness are 0.440 cm and 0.128 cm, respectively.

Table 8 shows the BA loading pattern for the ATOM core. To flat radial power distribution, the biggest CSBA ball is loaded into the inner zone, zone I, while smaller balls are placed in the outer regions. The CSBA design in the center FA is the same with that in zone III. On the other hand, only single design of the B_4_C DiBA is used for the simplicity of the core design. Thickness of the B_4_C disk is 40 micron, while the B_4_C disk radius is 0.22 cm. The total BA height, including B_4_C and Zr-4 clad, is 120 micron, resulting in an axial active core height of about 202 cm, about 2% higher than its original value^7^.

**Table 6:** Fuel shuffling scheme of the two-batch TOP ATOM core.

Zone I		Zone II		Zone III	
Fresh	Burned	Fresh	Burned	Fresh	Burned
C2	A3	B2	A2	B3	H1
D2	B4	D4	D5	C4	E2
D3	C5	F4	F5	G4	E5
E3	C3	H2	K2	H3	F1
F2	H4	K1	E4		
F3	G5				
G1	G3				
G2	K3

**Table 7:** CR design for the two-batch TOP ATOM core.

Parameters	Value
CR radius (cm)	0.51674
CR gap radius (cm)	0.52055
CR clad radius (cm)	0.56754
Inner tube radius (cm)	0.63979
Outer tube radius (cm)	0.70000
Mn rod radius (cm)	0.44000
Inconel-625 clad thickness (cm)	0.12800
Shutdown rod material	90 w/o B-10 B_4_C
Regulating rod material (type 1)	Natural B_4_C
Regulating rod material (type 2)	Inconel-clad Mn
MS material	Inconel-clad Mn

### Numerical results and discussion

To investigate the neutronic performance of the ATOM core, the Monte Carlo Serpent 2 code has been used with the library ENDF/B-VII.1^14^. The number of active and inactive cycles are 300 and 100, respectively, with 300,000 histories per cycle. The associated uncertainty of the multiplication factor (*k*_*eff*_) is about 10 pcm. In the Serpent calculation, the effective temperature of the fuel is fixed at 840K^27^ and a linearly varying axial coolant temperature is considered with an average temperature of 575 K. Temperatures at CZP and HZP are 294 K and 575 K, respectively. Note that the ATOM core adopts a constant average coolant temperature strategy during nominal operation.

**Figure 10:**
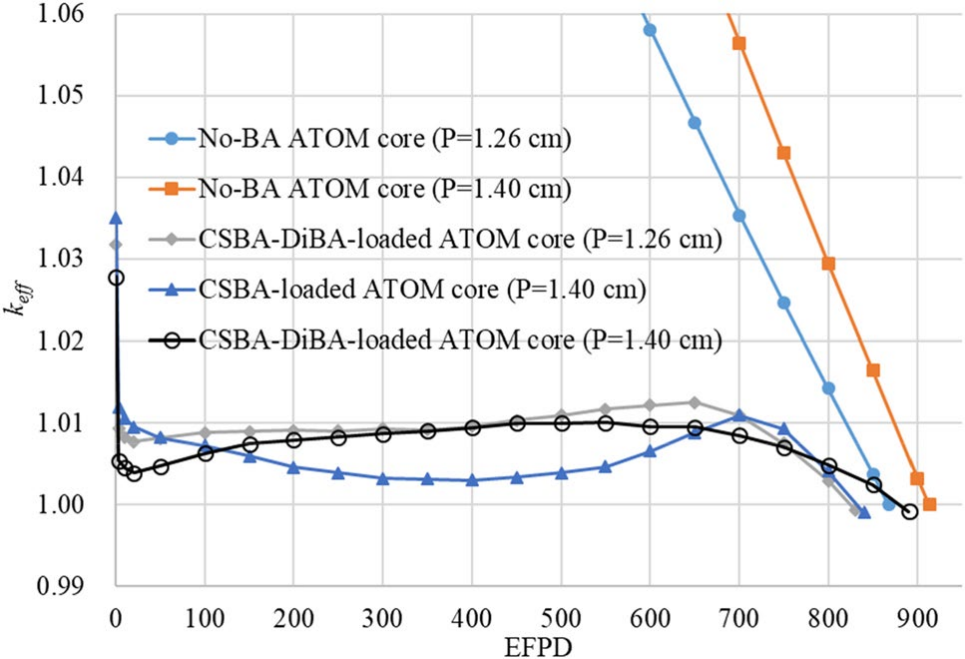
The excess reactivity of several equilibrium cores.

The neutronic performances of several equilibrium cycles are shown in Fig. 10 and Table 9. It can be observed that the excess reactivity can be very successfully minimized by using the CSBA and DiBA technologies, and the cycle length of the TOP cores is significantly enhanced compared to that of the standard FA design with the pin pitch of 1.26 cm. One can see that the cycle length is extended by ~ 73 effective full power days (EFPDs) in the CSBA-DiBA-loaded core. On the other hand, it is also noteworthy that the CSBA-DiBA core provides about 60 EFPDs longer cycle length than the CSBA-only one, which is clearly due to quite smaller reactivity penalty due to residual gadolinium in the CSBA and DiBA hybrid case. As a result, Table 9 confirms that the fuel discharge burnup is similarly increased with the TOP-based. In all candidate equilibrium cores, the reactivity swing is only around 1000 pcm and this is very favorable for the SBF operation since the criticality can be easily controlled by using weak CRs such as MS in Table 10 without causing local power peaking issue.

**Table 8:** The BA design for the TOP ATOM core.

Zone	CSBA radius	DiBA radius	DiBA height	Fuel enrichment
I	1.45 mm	2.2 mm	40 micron	4.95 w/o
II	1.25 mm			4.95 w/o
III	1.18 mm			4.95 w/o
Center FA	1.18 mm			3.00 w/o

**Figure 11:**
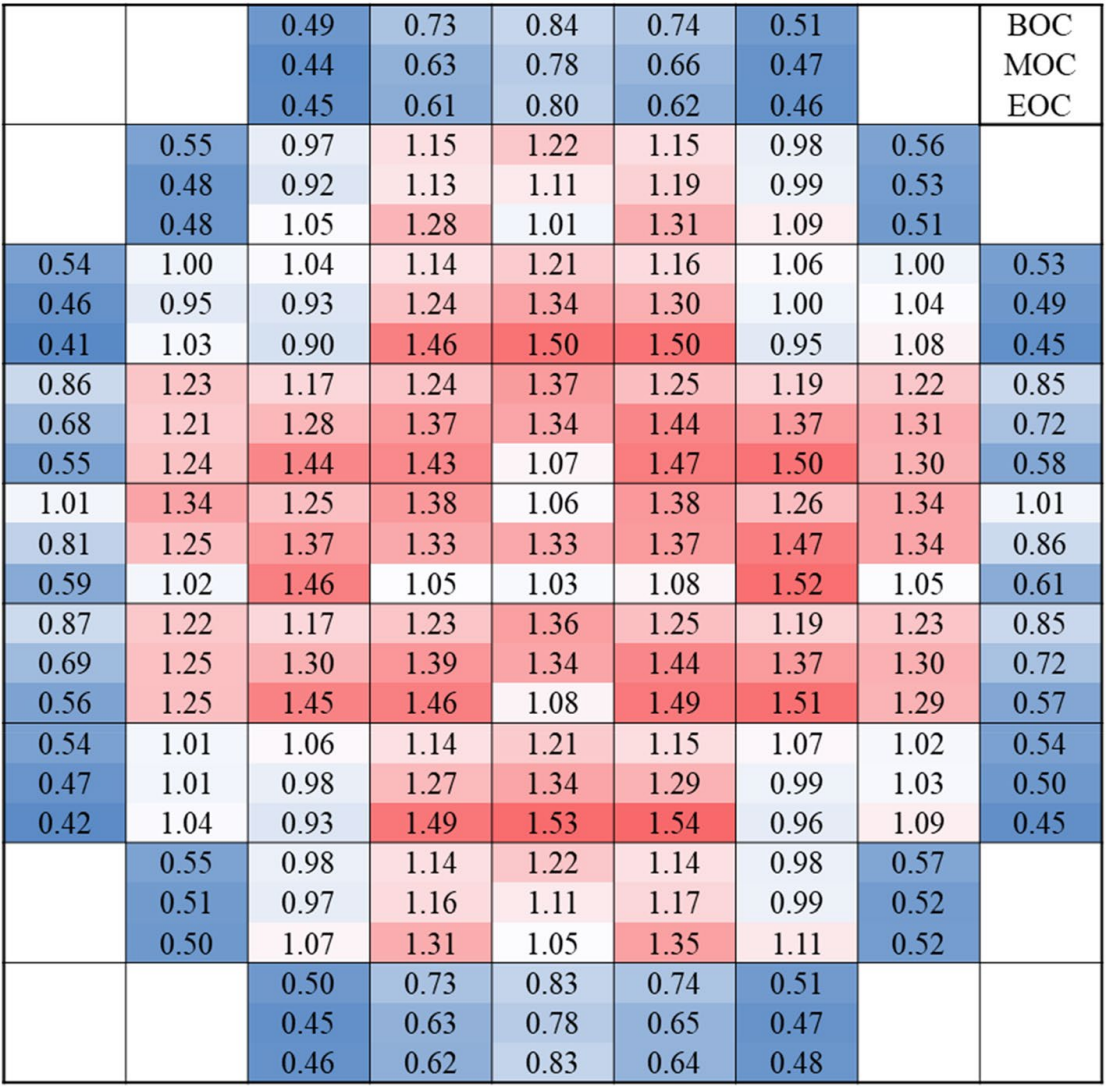
Radial assembly-wise power distribution of the TOP ATOM core.

**Figure 12:**
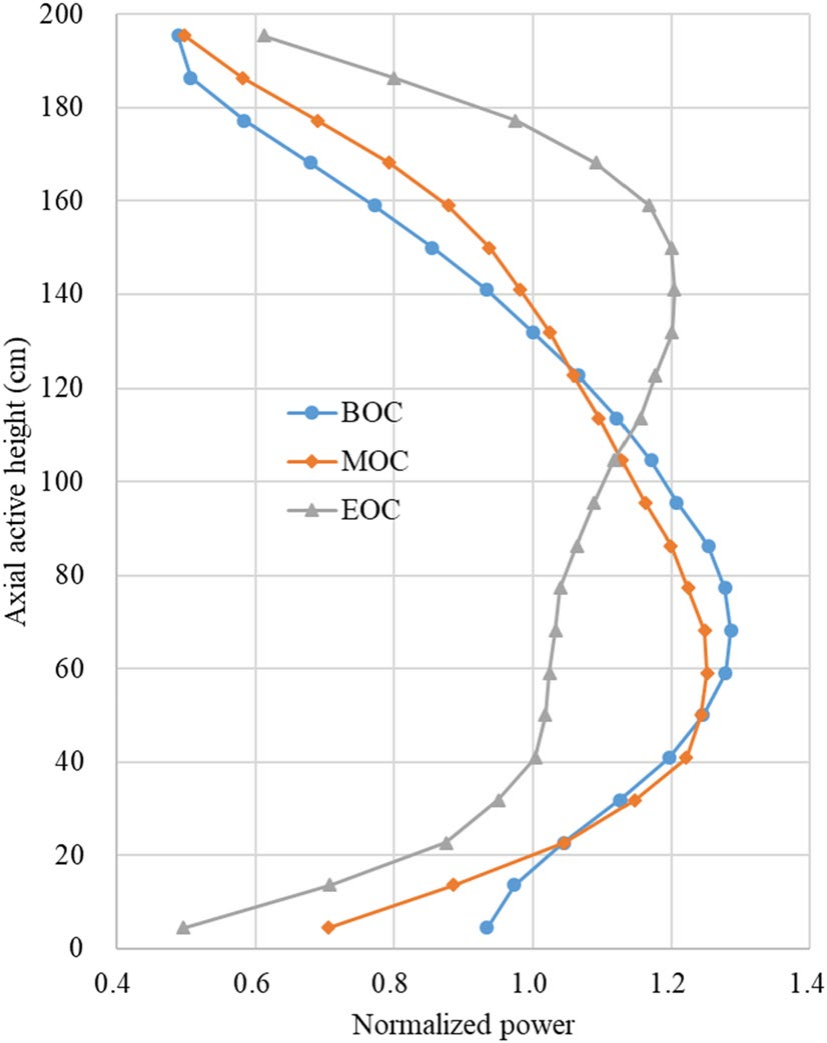
Core-wise axial power distribution of the TOP ATOM core.

For the CSBA-DiBA TOP-based core, assembly-wise radial and core-wise axial power profiles are depicted in Figs. 11 and 12, respectively. It is observed that the radial peaking factor is rather small, ~ 1.5 at the EOC condition, even though the fuel shuffling is based on a low leakage scheme. The axial power profile is clearly bottom-skewed at the BOC due to the SBF condition and it becomes slightly top-skewed at EOC, and the axial peaking factor is less than 1.3 at any condition. The associated uncertainties for radial and axial powers are 0.5% and 0.2%, respectively. Appropriate axial zoning of the BAs can be easily adopted to alleviate the skewed power distributions.

Table 10 shows temperature coefficients of the CSBA-DiBA TOP-based ATOM core for various conditions. In the temperature coefficient evaluation, the maximum associated uncertainty of FTC and MTC is 0.08 pcm/K and 0.30 pcm/K, respectively. Both MTC and fuel temperature coefficient (FTC) are clearly negative at any conditions, assuring the inherent safety of the ATOM core. One should note that the HFP MTC is always strongly negative and its variation is only about -3 pcm/K between BOC and EOC* due to the TOP design, while it is about − 14 pcm/K with the standard pitch design^7^. It is important to note that there is no concern about ‘too much negative’ MTC in the ATOM core due to the TOP concept, which will improve its operational safety and flexibility. Such appropriately negative MTC values are favorable for the autonomous operation of the reactor as it minimizes the coolant temperature variation^22^.

**Table 9:** Neutronic performance of the ATOM cores.

Case	Reactivity swing (pcm)	Cycle length (EFPD)	Discharge burnup (GWd/tU)
No-BA (Standard)	–	868	44.1
No-BA (TOP)	–	913	46.5
CSBA-DiBA (Standard)	1239	817	42.8*
CSBA-only (TOP)	1040	830	44.3*
CSBA-DiBA (TOP)	988	890	45.6*

*Without central FA.

**Table 10:** Temperature coefficients of the TOP ATOM core.

Condition	MTC (pcm/K)	FTC (pcm/K)
HFP-BOC	− 38.60	− 2.20
HZP-BOC	− 33.49	− 2.54
HFP-EOC*	− 41.03	− 2.34
HZP-EOC*	− 35.05	− 2.64

EOC*: @ 700 EFPD near EOC.

The (N-1) shutdown margin was evaluated assuming a CEA is stuck out at the CZP condition and Table 11 shows the result. The statistical uncertainty of the rod worth in Table 11 is less than 10 pcm. The shutdown evaluation is evaluated at both CZP-BOC and CZP-EOC* conditions without any Xe in the core. Note that excess reactivity is noticeable at EOC* while core characteristics are very similar to those of EOC. It is very clear that the proposed pseudo CR pattern provides enough N-1 shutdown margin to assure sub-criticality at the CZP condition.

In the ATOM core, both MS and RR are to be used for reactivity control during normal operations and the HFP excess reactivity is supposed to be compensated by the MS only at the Xe-equilibrium condition. Table 12 lists the reactivity worth of the MS and regulating CR at both HZP and HFP conditions without Xe. The maximum associated uncertainties in the rod worth are about 7.0 pcm. The MS rod worth ranges from 901 to 933 pcm, which is sufficient to compensate for the burnup reactivity swing during the equilibrium cycle. It is also noted that all excess reactivity at HZP without Xe can be controlled by using both MS and regulating rods.

**Table 11:** Shutdown margin of the TOP-based ATOM core.

Scenario (CZP)	BOC, No Xe		EOC*, No Xe	
	*k*_*eff*_	Rod worth (pcm)	*k*_*eff*_	Rod worth (pcm)
ARO (all-rod-out)	1.08376	-	1.09203	-
ARI (all-rod-in)	0.95708	12,213	0.92854	16,122
N-1 (E1)**	0.96599	11,250	0.94551	14,190
N-1 (F2)**	0.97976	9,794	0.97797	10,680
N-1 (E3)**	0.97931	9,842	0.98062	10,403
N-1 (F4)**	0.98989	8,750	0.97830	10,646
N-1 (G3)**	0.95798	12,115	0.93243	15,674

EOC*: @ 700 EFPD near EOC, **Stuck rod position.

**Table 12:** Mechanical shim and regulating rod worth for the ATOM core.

No Xenon	Rod worth (pcm)	
	BOC	EOC*
All-MS- and RR**-In (HZP)	3,495	3,892
All-MS-In (HFP)	901	933

**Figure 13:**
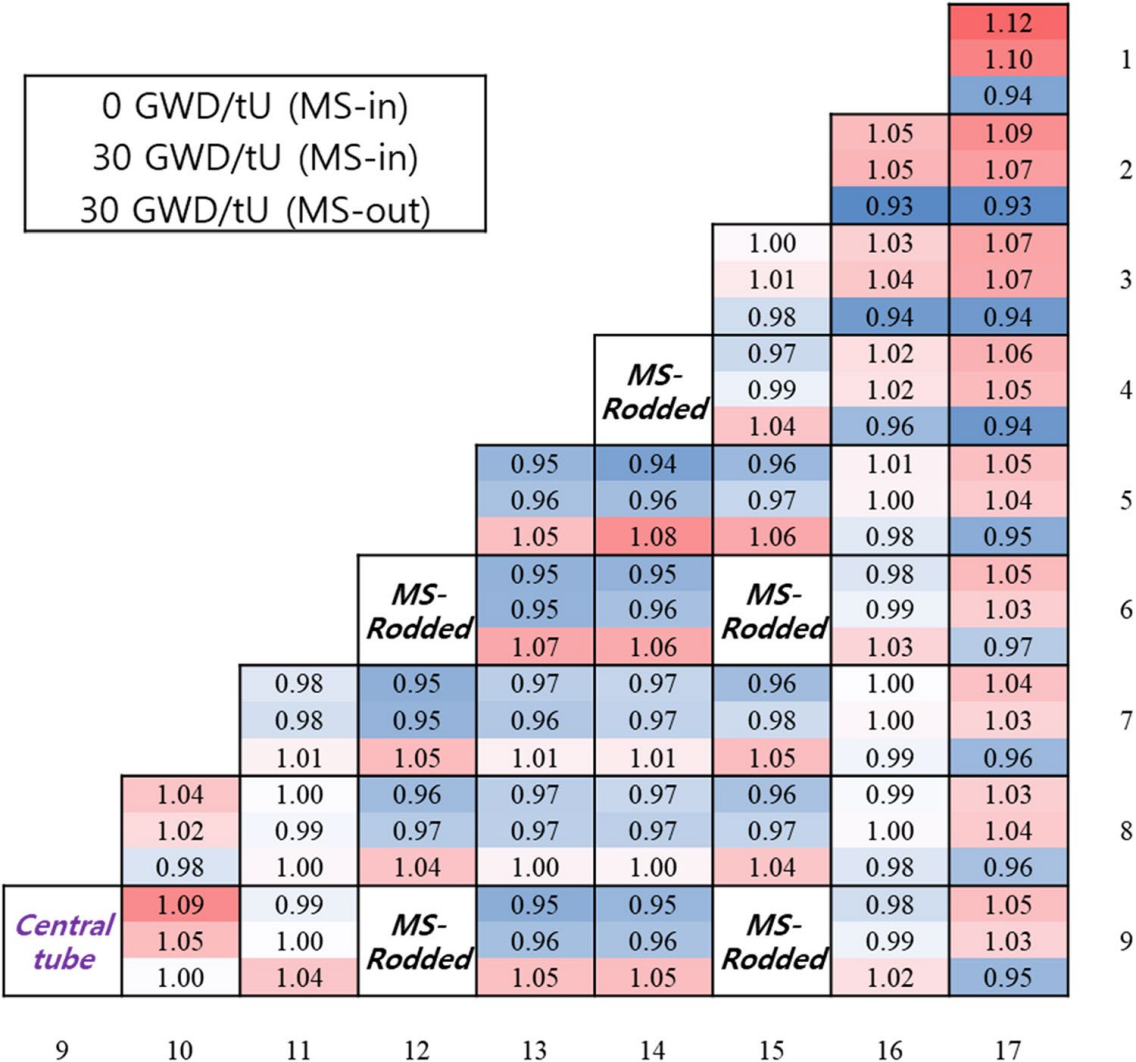
Impact of MS-rodded operation on local power distribution in FA.

It is recalled that MS rods should be rather fully inserted during the normal full power operation and they are to be gradually withdrawn near EOC for criticality control. The MS withdrawal near EOC should lead to a higher local power nearby guide tubes. To evaluate impact of the MS movement on the local power in a conservative way, a single MS-rodded FA was depleted to a high burnup of 30 GWD/tU and the MS rods were pulled out at 30 GWD/tU. Figure 13 shows the power distributions due to the MS maneuvering. In Fig. 13, uncertainty of the pin powers is about 0.3%. One can see that the pin power distribution is rather flat with a peaking factor of 1.12 at the FA corner even when all MS rods are fully inserted. It is also noted that the peak power is only about 1.07 near a guide tube when all MS rods are suddenly pulled out at 30 GWD/tU. Therefore, it is clear that the use of MSs is fully acceptable for the excess reactivity control in the SBF ATOM core.

## Conclusions and future works

The current work demonstrates that use of the TOP lattice clearly improves both cycle length and discharge burnup of the SBF SMR. Moreover, the TOP FA design also results in the more desirable MTC and FTC for inherent safety, power maneuvering, and autonomous operation of the SBF core, while the cold shutdown is guaranteed with a pseudo checker-board control pattern. On the other hand, the innovative CSBA and/or DiBA technologies enable a high-performance SBF design of the ATOM core in which the burnup reactivity swing is less than 1,000 pcm. As a result, the criticality control of the SBF core is achievable with a weak MS bank only and the consequent local power perturbation is quite marginal. Furthermore, it is shown that the use of B_4_C DiBA in the CSBA-DiBA hybrid case can significantly reduce the reactivity penalty due to residual gadolinium isotopes in the CSBA, enabling a quite longer cycle length. Overall, a high-performance SBF ATOM core has been successfully developed by utilizing the TOP lattice and the CSBA-DiBA hybrid designs. It is expected that the newly proposed TOP-based SBF core concept would be a new design paradigm for future high-performance PWRs.

The axial power distribution of the SBF ATOM core is rather bottom-skewed at the BOC condition due to the strongly negative MTC. An axial BA zoning will be implemented to achieve more favorable axial power shapes. In addition, further optimization on the CR pattern will be done to introduce a fully checker-board CR arrangement.

## Supplementary Information

Supplementary Information.

## Abbreviations

ATOM: Autonomous transportable on-demand reactor module
BA: Burnable absorber
BOC: Beginning of cycle
CASMRR: Center for autonomous small modular reactor research
CSBA: Centrally-shielded burnable absorber
CR: Control rod
CZP: Cold zero power
DiBA: Disk-type burnable absorber
EOC: End of cycle
FTC: Fuel temperature coefficient
HFP: Hot full power
H-to-D: Height-to-diameter
HTU: Hydrogen-to-uranium
HZP: Hot zero power
KAIST: Korea Advanced Institute of Science and Technology
MOC: Middle of cycle
MS: Mechanical shim rod
MTC: Moderator temperature coefficient
PWR: Pressurized water reactor
SB: Soluble boron
SBF: Soluble-boron-free
SSE: Self-shielding effect
SMR: Small modular reactor
TH: Thermal hydraulics
