# Supplementary material for: Truly-optimized PWR lattice for innovative soluble-boron-free small modular reactor
Source: Sci Rep. 2021 Jun 18;11:12891. doi: 10.1038/s41598-021-92350-5 (PMC8213724; doi:10.1038/s41598-021-92350-5)
Supplement: Supplementary file 1 — Supplementary Information. [file 41598_2021_92350_MOESM1_ESM.docx]

# Supplementary information

1. **The thermal hydraulics coupled neutronic analysis of the ATOM core**

Monte Carlo-deterministic hybrid two-step calculations are performed for the two-batch ATOM core analysis. Firstly, spatial homogenization of the FA is done by using Monte Carlo Serpent 2 [1] in conjunction with the nuclear library ENDF/B-VII.1. Then, a 3-D nodal code, COREDAX [2], performs the thermal-hydraulics (TH) coupled neutronic calculations. A wrapper script for the two codes and two-step Serpent-COREDAX calculation was verified and validated for the analysis of PWR [3].

It is assumed that the standard single channel analysis is applicable for the CSBA-DiBA-loaded design. The detailed (TH) properties of the core are tabulated in Table A-I. The number of radial meshes for fuel pin and cladding are 10 and 4, respectively. The net mass flow rate is determined so that the temperature rise is 35.7 K.

Table A-I: Thermal hydraulics conditions of the two-batch ATOM core

| Parameters | Target value | Unit |
| --- | --- | --- |
| No. of radial mesh for fuel pin/cladding | 10/4 |  |
| Net mass flow rate | 2,259 | kg/s |
| Gap heat transfer coefficient | 11,345 | W/m^2^K |
| Inlet coolant temperature | 558.0 | K |
| Outlet coolant temperature | 593.7 | K |

The comparison between the multiplication factors calculated by Serpent and Serpent-COREDAX is presented in Fig. A-1. The good agreement is witnessed at BOC and EOC conditions, however the multiplication factor calculated by Serpent-COREDAX is slightly underestimated during the cycle. The maximum reactivity difference is about 230 pcm.


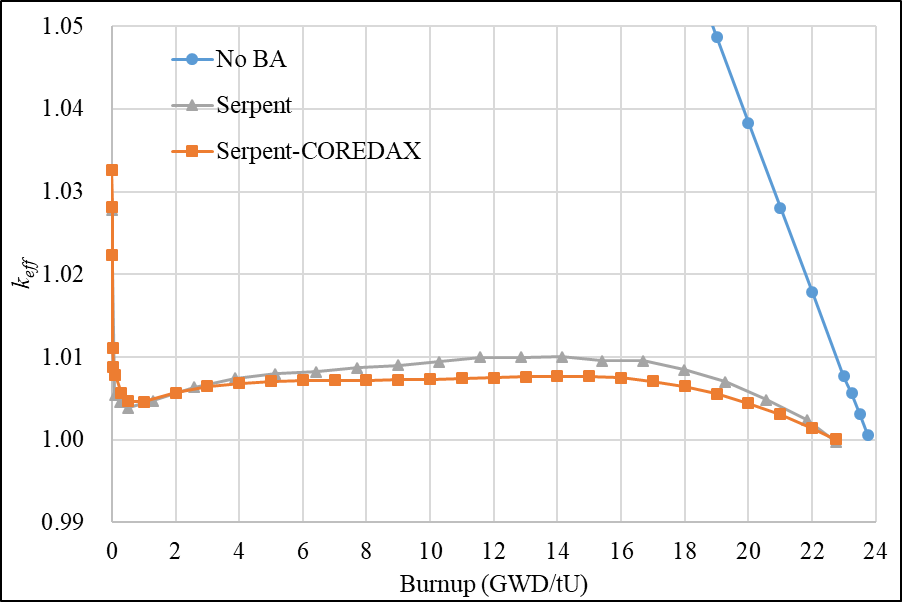


Figure A-1: Multiplication factor comparison between Serpent-COREDAX and Serpent calculations

On the other hand, the radial and axial power distributions calculated by Serpent-COREDAX are presented in Figs. A-2 and A-3. It can be seen that the TH-coupled neutronic analysis provides a bit more bottom-skewed axial power distribution at BOC compared to the Serpent-calculated one. This is because a uniform fuel temperature assumption is used in the Serpent-only calculation. Consequently, the COREDAX power distributions at MOC are quite different, which explains the underestimation of multiplication factor in the Serpent-COREDAX calculation. One notes that the EOC axial power distribution is quite similar to the Serpent-only case. On the other hand, the two codes show a good match in terms of radial power with about 3% relative error at the hottest FA. A largest relative error, about 11%, is found at the coldest FA near the reflector.


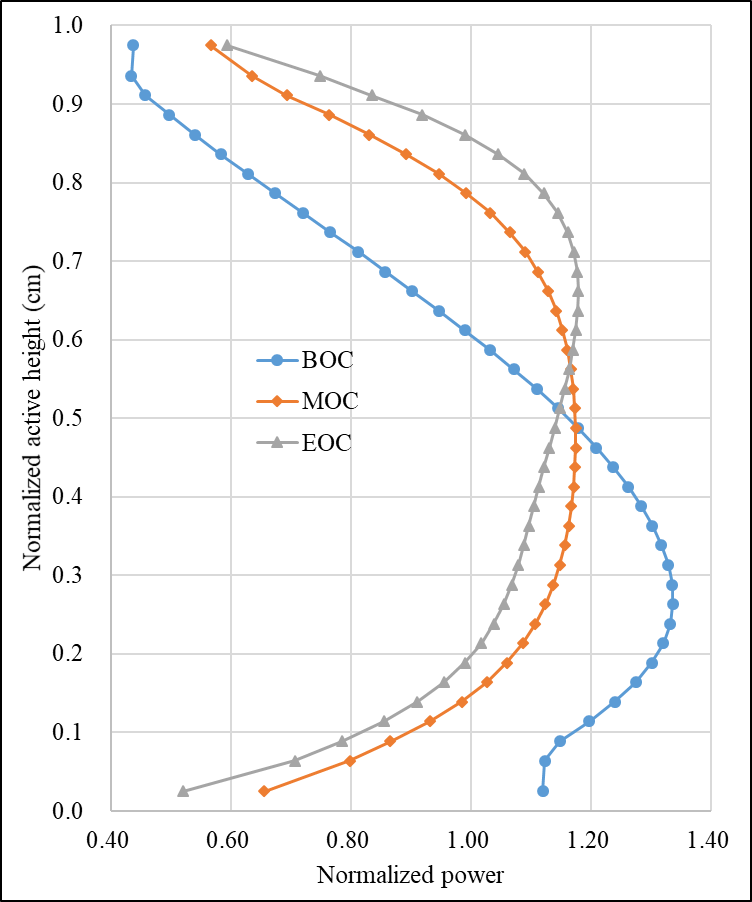


Figure A-2: Axial power distribution calculated by Serpent-COREDAX


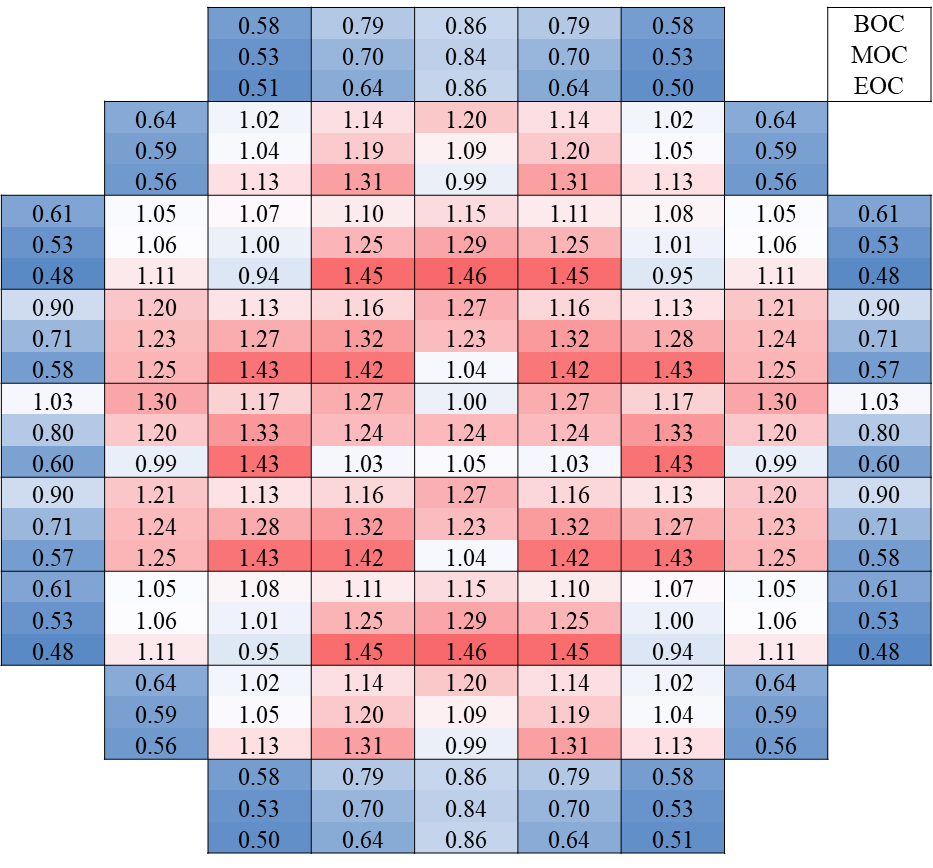


Figure A-3: Radial power distribution calculated by Serpent-COREDAX.

The axial core-average temperature distributions are shown in Fig. A-4. The fuel temperature closely follows the axial power distribution as expected. The maximum fuel temperature is about 793K at the BOC condition where the power is highly bottom-skewed. Meanwhile, the coolant temperature distribution is typical of PWR-type reactor and the coolant temperature rise is about 35.7 K.

Overall, the comparison between the Serpent-only and Serpent-COREDAX calculations implies that Serpent-only analyses in this work are quite acceptable in characterizing the ATOM core.


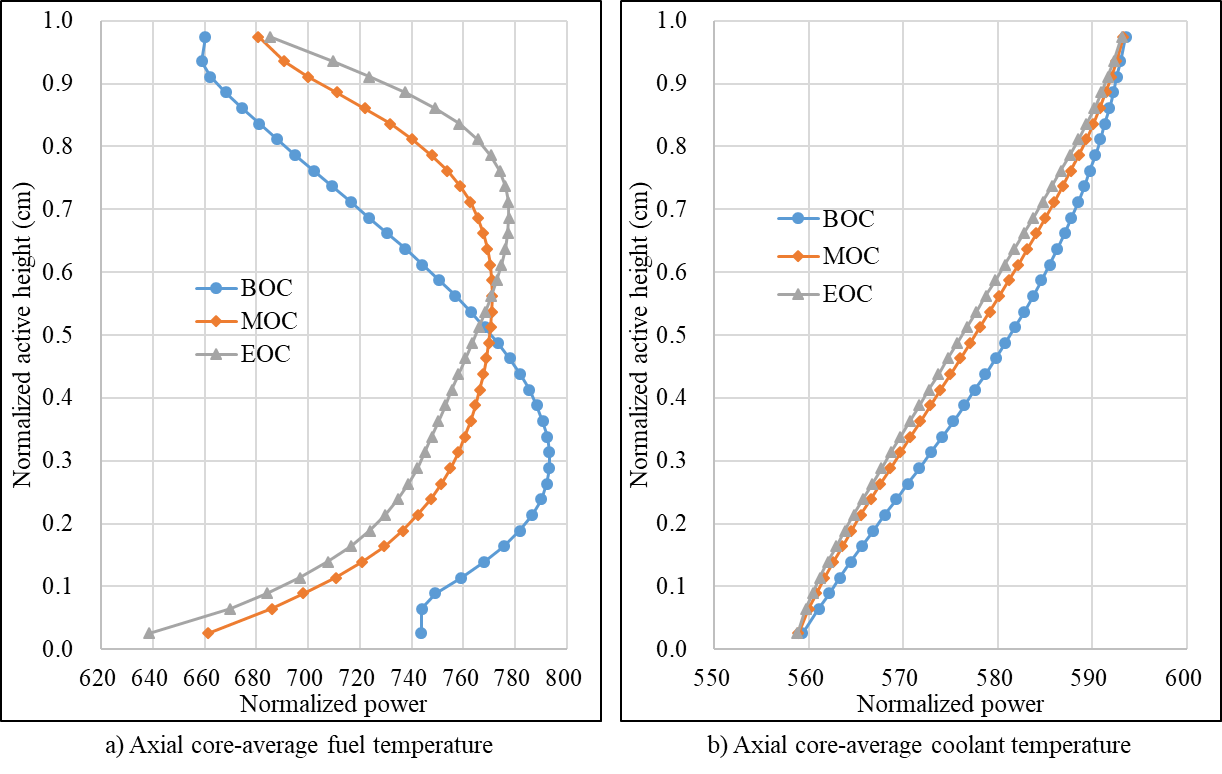


Figure A-4: Axial core-average temperature distribution

1. **The thermal and neutronic analyses of CSBA-DiBA fuel rod**

The insertion of DiBA between fuel pellets and CSBA in the center of pellet result in a significant material discontinuity in the axial direction, which may cause a noticeable difference in the axial power shape. To quantify the axial peaking, a fuel rod consisting of 200 CSBA-loaded fuel pellets is modelled with radial reflective and axial vacuum boundary conditions, in which 199 DiBAs are also installed in the 1P1D pattern. The detailed fuel rod problem is tabulated in Table B-I. As in the ATOM fuel rod, 5 cm BA cutback and 5 cm low-enriched UO_2_ are applied at both top and bottom of the BA-loaded fuel rod. Figure B-1 shows the detailed axial distribution of the CSBA-DiBA and No-BA fuel rods. It can be seen that the insertion of the DiBA and CSBA results in a saw-tooth power distribution. The power is strongly suppressed at both CSBA and DiBA locations. It can be observed that the saw-tooth behavior becomes more significant at the mid-plane, where the axial peaking is found. One should note that the magnitude of the fluctuation is about 7% and it is recalled that the non-uniform power profile due to CSBA should not cause any problems as discussed in Ref. [4]. Nevertheless, the peaking factor of the BA-loaded fuel rod is about 4% lower than that of the no-BA case due to BA cutbacks that cause two small power peaks at the top and bottom of the fuel rod. The associated uncertainty of the axial power is only 0.06% in this analysis.

Table B-I: Single fuel rod problem

| Parameter | Value |
| --- | --- |
| Number of fuel pellet per rod | 200 |
| Gd_2_O_3_ CSBA radius | 0.12 cm |
| Number of B_4_C 1P1D DiBA | 199 |
| B_4_C thickness | 40 micron |
| B_4_C radius | 0.22 cm |
| Pellet height | 1.0 cm |
| Pellet dish depth | 0.0287 cm |
| Pellet dish radius | 0.205 cm |
| Axial tally mesh | 0.2 cm |
| Histories | 500,000 |
| Number of active and inactive cycles | 250/100 |


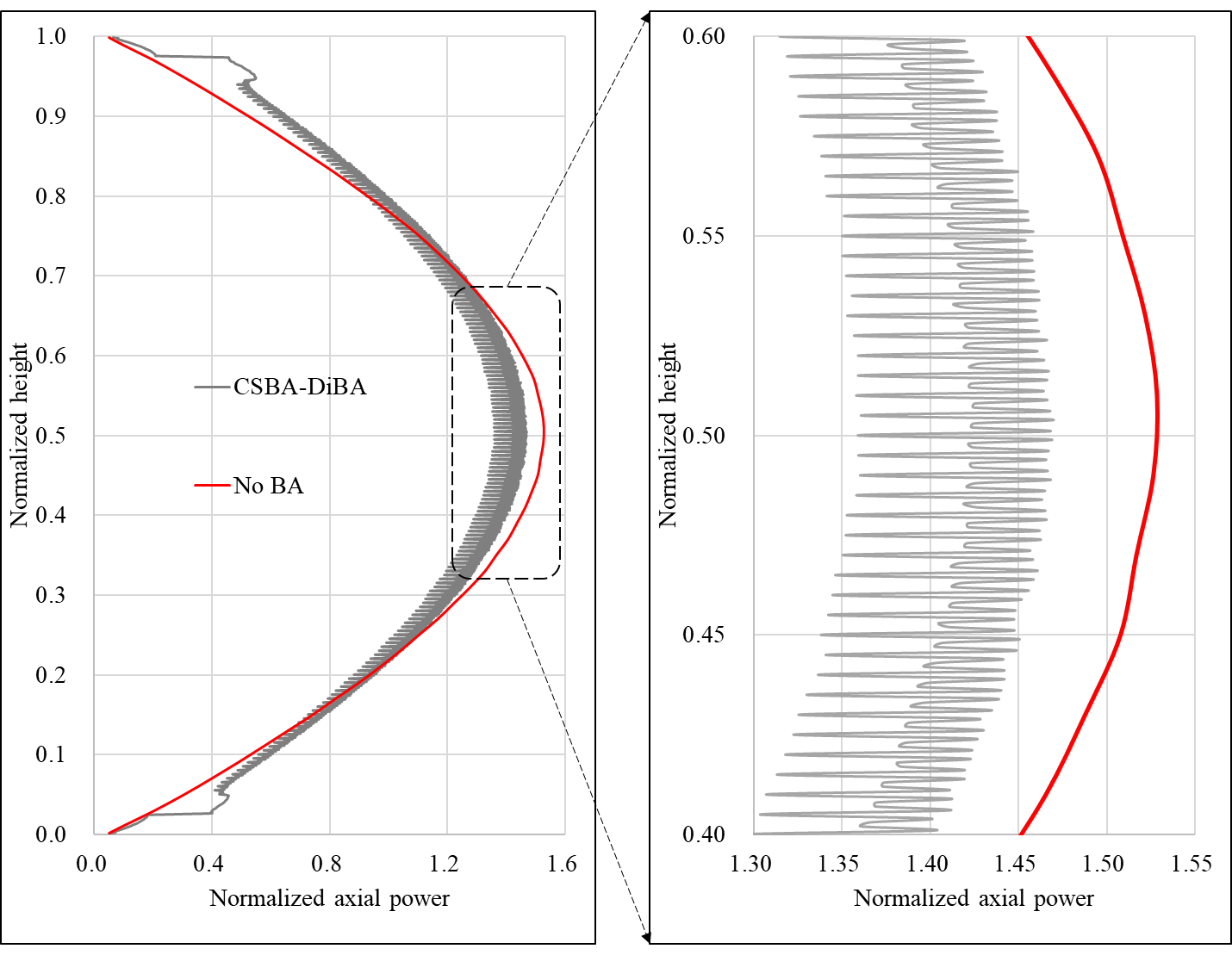


Figure B-1: Axial power distribution for the CSBA-DiBA and No-BA fuel rods

Thermo-mechanical analysis of the CSBA-DiBA-loaded fuel pellet was also performed for a fresh fuel condition to evaluate detailed temperature distribution in the fuel and BA regions. The numerical results are obtained by using the commercial code COMSOL^®^ [5]. The fuel pellet configurations and calculational conditions are given in Table B-2 and Fig. B-2. The specific power density of the ATOM core, 26 W/gU, is used and the outer surface temperature of fuel cladding is fixed at 618K as the boundary condition. In addition, it is assumed that the fission heat is only produced in the fuel region and the axial heat conduction is negligibly small compared to radial one, and thermal insulation is applied at both top and bottom of the fuel geometries. The relevant thermo-physical material properties under PWR condition are utilized from the references [6] [7] [8] [9]. In this evaluation, the thermal properties of the CSBA-DiBA-loaded fuel pellet is assumedly identical to those of the conventional UO_2_ fuel pellet and independent of the fuel burnup.

Table B-2: Design parameters of the CSBA-DiBA fuel pellet for COMSOL analysis

| **Parameter** | **Value** |
| --- | --- |
| Pellet radius | 0.4096 cm |
| Pellet dish depth | 0.0287 cm |
| Pellet dish radius | 0.205 cm |
| He gap thickness | 0.00915 cm |
| Fuel clad thickness | 0.0573 cm |
| 1-ball Gd_2_O_3_ CSBA radius | 0.145 cm |
| B_4_C thickness | 40 micron |
| B_4_C radius | 0.22 cm |
| DiBA clad thickness | 40 microns |
| DiBA clad outer radius | 0.4096 cm |
| Specific power density | 26 W/gU |
| Fuel pellet clad surface temperature | 618 K |

The radial temperature and axial centerline temperature of the three fuels are depicted in Fig. B-3 for the average power density. One notes that normal and BA-loaded fuels have similar radial temperature profiles in the fuel region. The normal fuel shows a relatively flat axial distribution with a small reduction near fuel dishes filled with helium gas. Meanwhile, it is clear that the DiBA suppresses the axial temperature rather strongly. The significant temperature gradient along the axial direction in the CSBA-DiBA pellets indicate that detailed dynamic thermos-mechanical analysis must be investigated to quantify the structural integrity of the fuel pellet under reactor condition. Such an analysis will be conducted in a separate study as it is beyond the scope of the paper. In general, the BA-loaded fuel pellets are comparable with the standard one in terms of maximum pellet temperature.

Figure B-4 presents the temperature profiles of the three fuels with a hypothetically high power density: the specific power density is three times higher than the average one. One can see that the peaking temperature of the three pellets are similar, about 2,450 K, and much lower than the melting temperatures of both UO_2_ (3,138K) and Gd_2_O_3_ (2690 K). Moreover, the maximum temperatures of B_4_C and Zir-4 in the CSBA-DiBA fuel are much lower than the melting temperatures of B_4_C (2,718 K) and Zir-4 (2,122 K). Overall, the CSBA and CSBA-DiBA fuels have a similar performance with the normal fuel in terms of peak temperature and it is expected that the DiBA will survive a very high power condition.


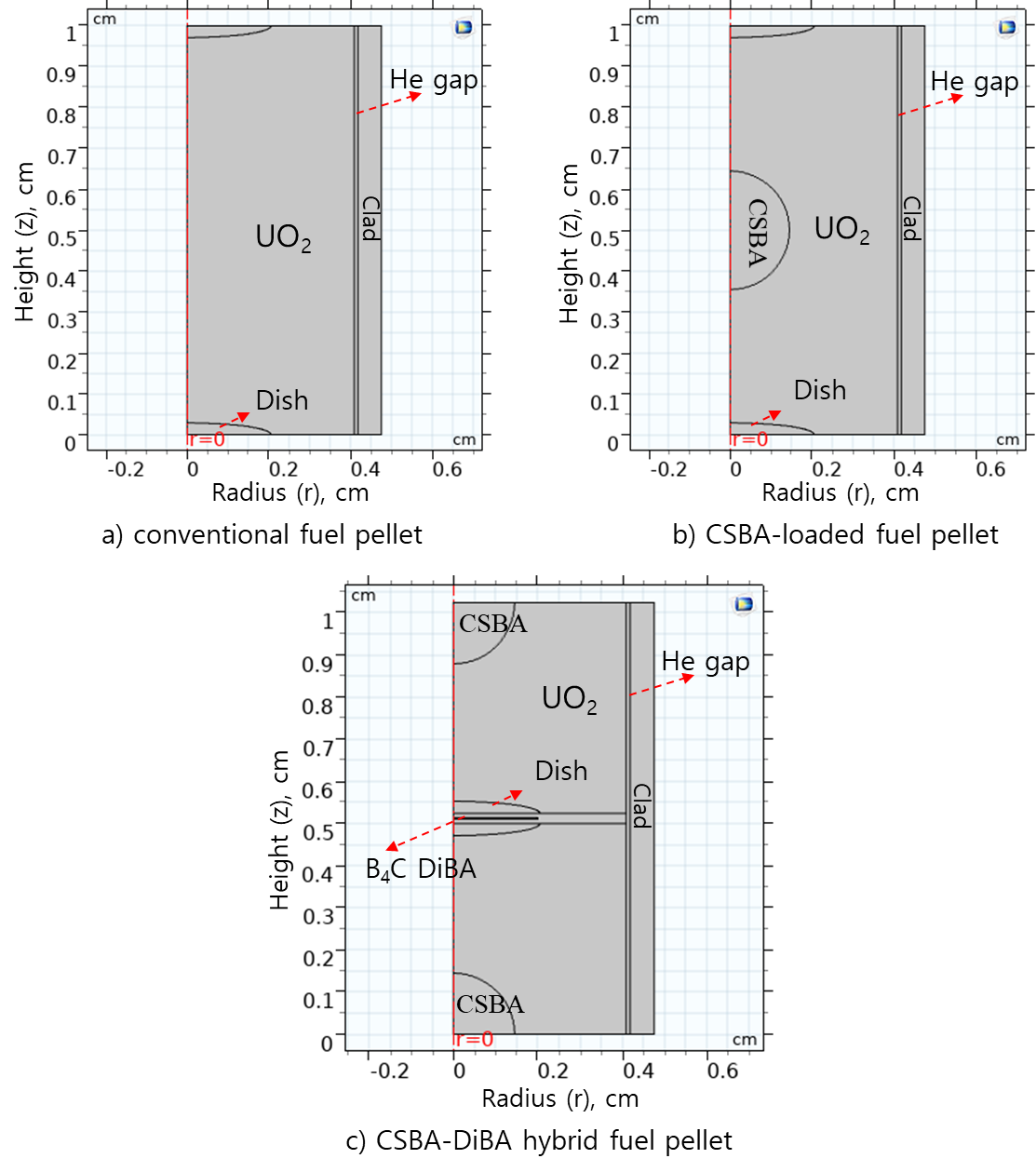


Figure B-2: Layout of the fuel geometries


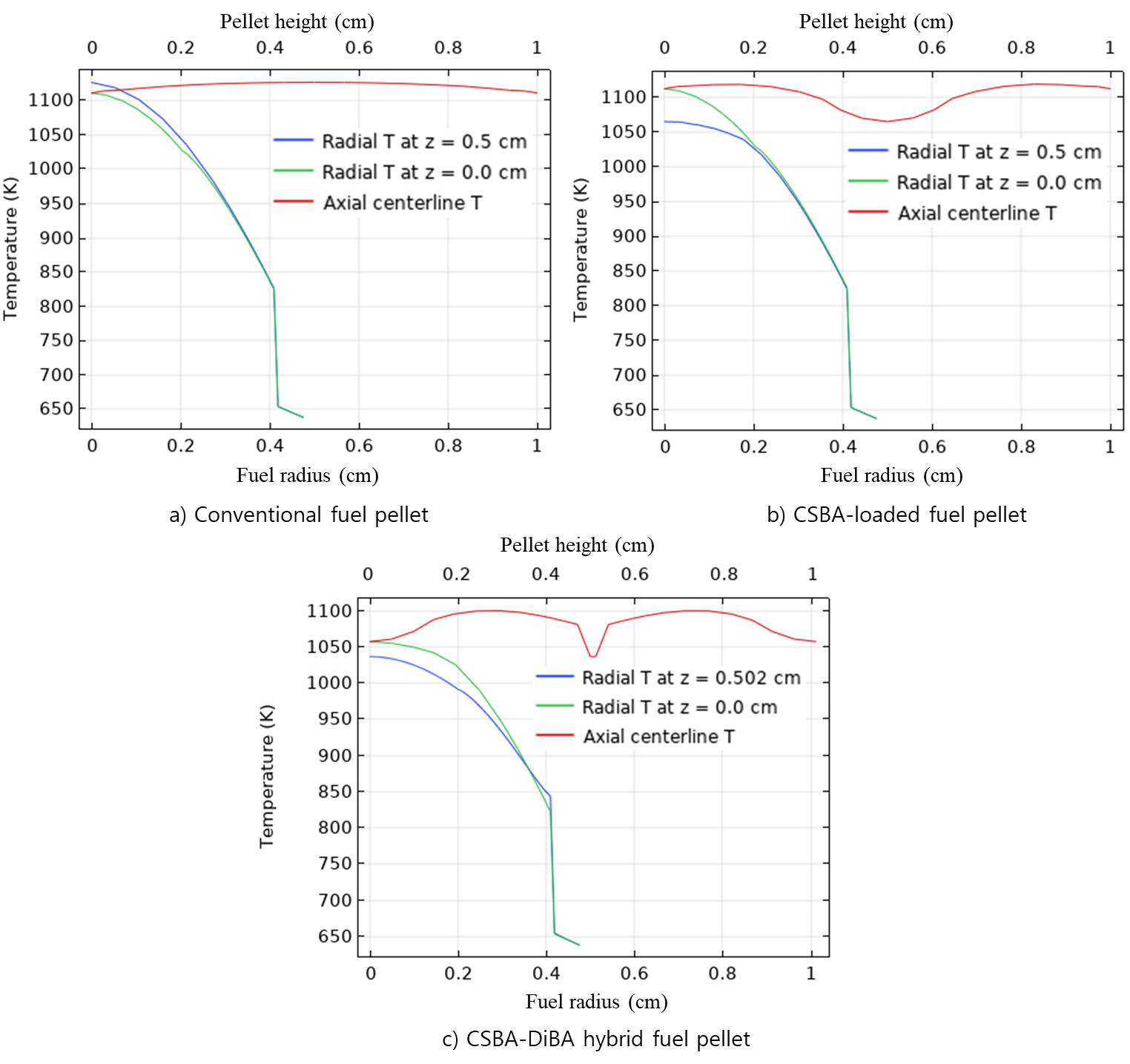


Figure B-3: Radial and axial temperature profiles in the fuel (average power density)


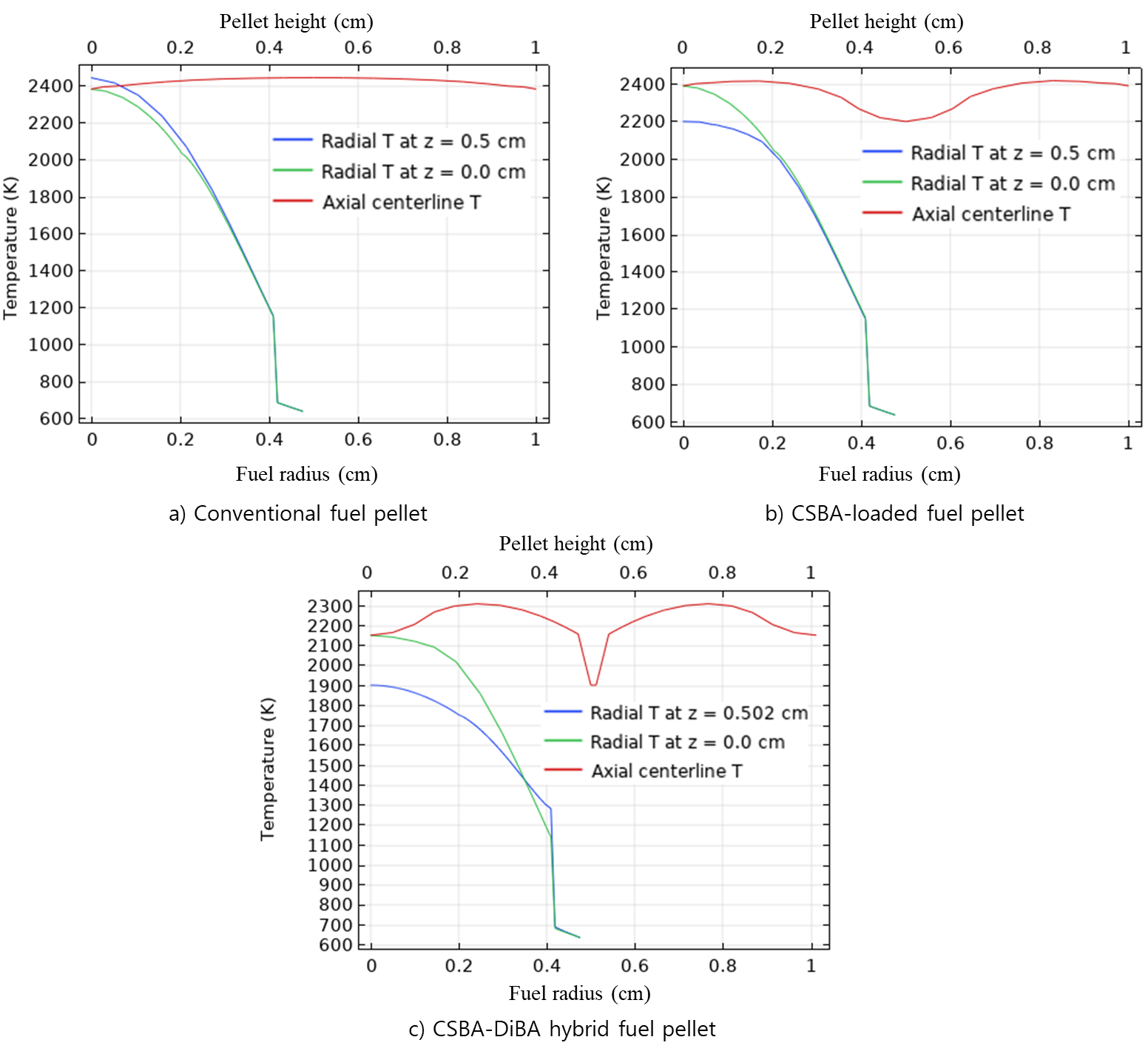


Figure B-4: Radial and axial temperature profiles in the hottest fuel (peak factor = 3.0)

1. **Checker-Board Control Rod Pattern with Extended Control Element Assembly**

The standard CEA for the 17x17 FA comprises a cluster of 24 control rods (24 fingers) which are inserted into 24 guide tubes. To enhance the shutdown margin, an extended shutdown CEA is introduced in the ATOM core, in which the number of finger per extended shutdown CEA is 36. The additional control rods are inserted into the neighboring FAs. A checker-board CR pattern utilizing the extended CEA is shown in Fig. C-1.

**
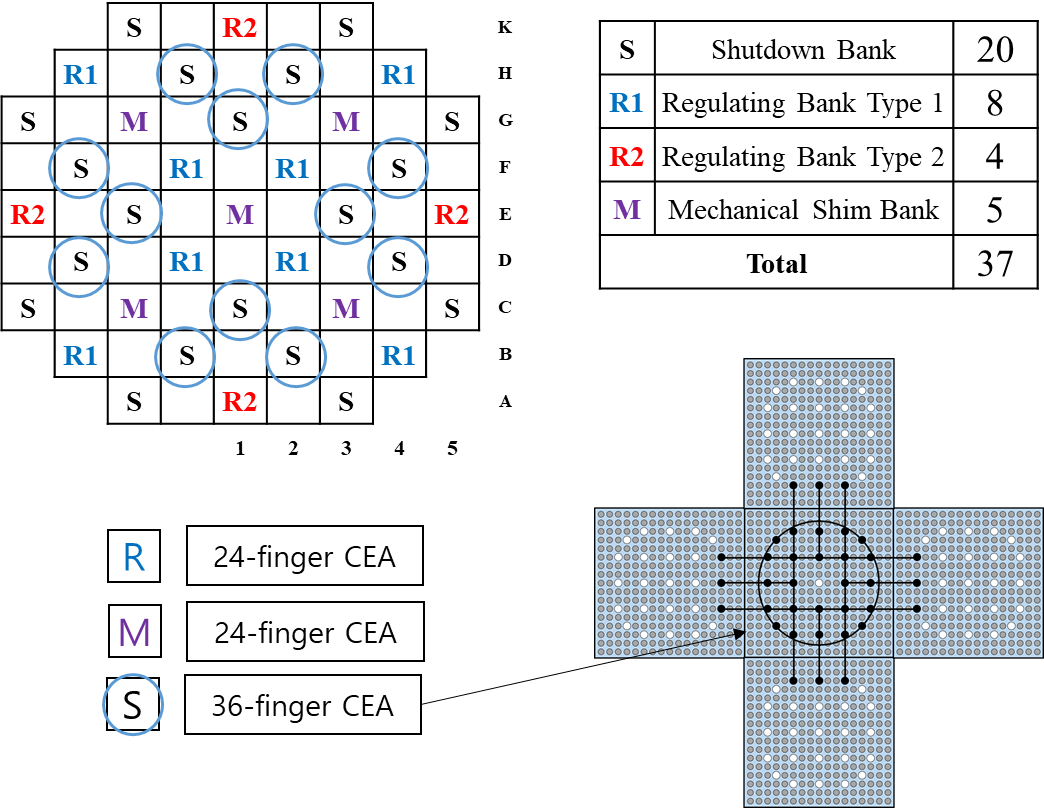
**

Figure C-1: The checker-board CR pattern with extended CEAs

The shutdown margin of the checker-board CR pattern is evaluated at the CZP condition by using the Serpent code and the numerical result is tabulated in Table C-1. The associated uncertainty of the rod worth is less than 10 pcm. The shutdown margin evaluation is also determined at both BOC and EOC* conditions without any Xe in the core. One can see that the proposed checker-board CR pattern with the extended CEAs provides sufficient N-1 shutdown margin to maintain the core at sub-critical state at the CZP condition.

Table C-1: Shutdown margin of the TOP-based ATOM core

| Scenario  (CZP) | BOC, No Xe | | EOC*, No Xe | |
| --- | --- | --- | --- | --- |
|  | *k_eff_* | Rod worth (pcm) | *k_eff_* | Rod worth (pcm) |
| ARO (all-rod-out) | 1.08376 | - | 1.09203 | - |
| ARI (all-rod-in) | 0.92819 | 15,465 | 0.90068 | 19,454 |
| N-1 (E1)** | 0.94444 | 13,612 | 0.92740 | 16,256 |
| N-1 (F2)** | 0.95805 | 12,107 | 0.95609 | 13,020 |
| N-1 (E3)** | 0.95991 | 11,904 | 0.96211 | 12,365 |
| N-1 (F4)** | 0.98351 | 9,405 | 0.98566 | 9,881 |
| N-1 (G3)** | 0.94537 | 13,507 | 0.92422 | 16,626 |
| N-1 (H2)** | 0.98087 | 9,679 | 0.98692 | 9,753 |
| N-1 (H4)** | 0.93974 | 14,141 | 0.92522 | 16,510 |
| *EOC*: @ 700 EFPD near EOC, **Stuck rod position* | | | | |

# References

| [1] | J. Leppänen, M. Pusa, T. Viitanen, V. Valtavirta and T. Kaltiaisenaho, "The Serpent Monte Carlo code: status, development and applications in 2013," *Annals of Nuclear Engergy,* vol. 82, pp. 142-150, 2015. |
| --- | --- |
| [2] | B. Cho, S. Yuk, N. Z. Cho and Y. Kim, "User's manual for the rectangular three‐dimensional diffusion nodal code COREDAX‐2 version 1.8," KAIST, Daejeon, Korea, 2016. |
| [3] | M. S. Yahya and Y. Kim, *A New Approach for a High-Performance Soluble-Boron-Free PWR core,* PhD Thesis, Daejeon: KAIST, 2016. |
| [4] | H. Kim and Y. Kim, "Unstructured Mesh–Based Neutronics and Thermomechanics Coupled Steady-State Analysis on Advanced Three-Dimensional Fuel Elements with Monte Carlo Code iMC," *Nuclear Sience and Engineering,* pp. 1-14, 2021. |
| [5] | "COMSOL Multiphyiscs v.5.4. www.comsol.com.," COMSOL AB, Stockholm, Sweden, 2018. |
| [6] | Q. Mistarihi, F. B. Sweidan and H. J. Ryu, "Thermo-physical properties of bulk Gd2O3 for fuel performance analysis of a lumped burnable absorber fuel design," in *Transaction of the Korean Nuclear Society*, Gyeongju, Korea, Oct. 25-27, 2017. |
| [7] | J. K. Fink, "Thermophysical properties of uranium dioxide," *Journal of Nuclear Material,* vol. 279, pp. 1-18, 2000. |
| [8] | F. B. Sweidan and H. J. Ryu, "Composite Material Properties Simulation for the Fuel Performance evaluation of Gadolinia-cored UO2 Fuel," in *Water Reactor Fuel Performance Meeting*, Jeju, Korea, 2017. |
| [9] | IAEA-TECDOC-1496, "Thermophysical properties database of materials for light water reactors and heavy water reactors," IAEA, final report, 1999-2005. |
